# The oncogenic properties of the *EWSR1::CREM* fusion gene are associated with polyamine metabolism

**DOI:** 10.1038/s41598-023-31576-x

**Published:** 2023-03-25

**Authors:** Heidi Kaprio, Arafat Siddiqui, Lotta Saustila, Vanina D. Heuser, Maria Gardberg

**Affiliations:** 1 Department of Pathology, Turku University Hospital, Kiinamyllynkatu 10 D, Turku, Finland; 2 Department of Obstetrics and Gynecology, Turku University Hospital, Turku, Finland; 3 Institute of Biomedicine, University of Turku, Turku, Finland

**Keywords:** Oncogenes, Cancer genetics, Cell division, Cell migration, Senescence

## Abstract

The *EWSR1::CREM* fusion gene, caused by a chromosomal translocation t(10;22)(p11;q12), has been discovered in divergent malignancies, ranging from low-grade to highly malignant cancers. The translocation gives rise to a chimeric protein, EWSR1::CREM. The molecular mechanisms behind the oncogenic properties of the EWSR1::CREM protein have not previously been systematically characterized. In this study, we performed transcriptional profiling of the melanoma cell line CHL-1, with depletion of endogenous EWSR1::CREM protein using siRNA mediated knockdown. We found that the expression of 712 genes was altered (Log2 fold-change ≥ 2). We performed pathway analysis to identify EWSR1::CREM mediated pathways and cell studies to examine functional differences brought upon by the knockdown. Altered pathways involved cell cycle and proliferation, this was further validated by the cell studies where cell migration was affected as well. Among the target genes with the greatest downregulation, we discovered ODC1—a well-established oncogenic enzyme that can be pharmacologically inhibited and is essential for polyamine synthesis. We found that the main effects seen upon EWSR1::CREM knockdown can be reproduced by directly silencing ODC1 expression. These findings provide novel insights into pathogenesis of tumors harboring a *EWSR1::CREM* fusion gene, hopefully facilitating the development of novel therapeutic strategies.

## Introduction

**Figure 1:**
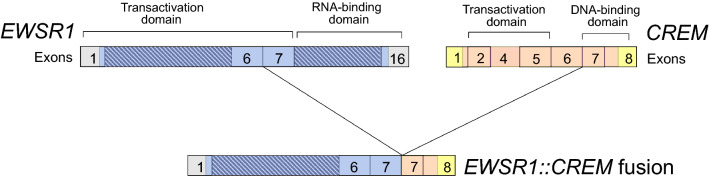
A schematic picture of the *EWSR1::CREM* fusion gene. Based on the work of Giacomini et al.^3^.

Cancer-associated gene fusions include essential driver mutations encoding abnormal chimeric transcription factors. Recurrent gene fusions involving the EWS RNA binding protein 1 (previously Ewing sarcoma breakpoint region 1, EWSR1) and the CREB family of transcription factors cAMP responsive element binding protein 1 (CREB1), activating transcription factor 1 (ATF1) and cAMP responsive element modulator (CREM) have been found in several histologically and phenotypically dissimilar malignancies, such as mesothelioma^1^, clear cell sarcoma^2^ and melanoma^3^. The most common fusion involving *EWSR1* and the *CREB* family is *EWSR1::ATF1*^4^, *EWSR1::CREM* (Fig. 1) is as common as *EWSR1::CREB1*, but far less studied. The prevalence of the *EWSR1::CREM* fusion gene in cancer has been estimated at 0.05%^4^. Of the 19.2 million new cancer cases per year worldwide, this would mean annually nearly 10,000 new patients affected^5^.

Fusion genes involving *EWSR1* typically encode aberrant transcription factors that alter gene expression. *EWSR1* provides a powerful transcriptional activation domain, while the transcription factor provides DNA binding. The fusion protein is usually transcriptionally highly active, altering gene expression levels and target gene specificity may be changed^6^. Despite this knowledge, the transcriptional activities of the fusion protein EWSR1::CREM are largely unexplored. The melanoma cell line CHL-1 is to our knowledge the only cell line harboring this mutation and expressing the fusion protein^3^, contributing means for such investigation.

In this study, we characterize the transcriptomic activities of the EWSR1::CREM chimeric protein in the CHL-1 cell line, using siRNA mediated knockdown of expression. Herein, we confirm that pivotal oncogenic pathways maintained by EWSR1::CREM involve cell replication. Loss of EWSR1::CREM protein is accompanied by downregulation of a multitude of genes, some of which are predicted target genes of CREM. Importantly, we identify that loss of ornithine decarboxylase (ODC1), an enzyme with critical involvement in polyamine synthesis, as an important mediator of the phenotype observed upon EWSR1::CREM knockdown.

## Materials and methods

### Cell culture

The commercially available cell lines CHL-1 (human melanoma), HEK-293 (human embryonic kidney), PC-3 (human prostate carcinoma), and WM-164 (human melanoma) were obtained from American Type Culture Collection (Manassas, VA, USA). All the cell lines were maintained in DMEM supplemented with 10% fetal bovine serum 5 mM ultraglutamine and 100 U/ml penicillin–streptomycin (Gibco; Carlsbad, CA, USA). ). The cells were regularly tested for mycoplasma contamination using MycoAlert® Mycoplasma Detection Kit (Lonza; Basel, Switzerland).

### Knockdown experiments

The *CREM* and *ODC1* transcripts were knocked down using 50 nM of human CREM small interfering RNA (siRNA) (sc-37700, Santa Cruz Biotechnology; Dallas, TX, USA) and ODC1 siRNA (s9823, Silencer Select ®, Ambion). A mix of three different non‐targeting siRNA (sc-37007, sc-44230, and sc-44231, Santa Cruz Biotechnology) was used as control. Cells were transfected using Dharmafect 4 transfection reagent (Dharmacon Research; Lafayette, CO, USA) according to the manufacturer’s instructions. The knockdown efficacy was examined 48 h after transfection by western blotting. The experiments were repeated at least three times.

### Western blotting

Cells were harvested and lysed with M-PER Mammalian Protein Extraction Reagent (Thermo Fisher) supplemented with protease inhibitors (Roche). Protein concentrations were measured using BioRad Bradford protein assay (Bio-Rad; Hercules, CA, USA) according to manufacturer's instructions. Equal amounts of proteins in Laemmli buffer were separated on 4–20% polyacrylamide PROTEAN® TGX™ Precast Protein Gels (Bio-Rad) and transferred to 0.2 µm nitrocellulose membrane using the TRANS-Blot Turbo Transfer System (Bio-Rad). Membranes were blocked with 5% dry milk in Tris-buffered saline (TBST) and probed overnight with primary antibodies diluted in the same solution. Our western blot membranes were cropped and incubated with different antibodies or the membranes were probed and reprobed after stripping. All original blots or replicates can be found in the Supplementary information—Original blots. Primary antibodies used in the western blotting were: mouse monoclonal anti-CREM (1:1000; clone 3B, Novusbio; Littleton, CO, USA), mouse monoclonal anti-CREB (LB9,1:1000, Abcam), mouse monoclonal anti-ODC1 (1:1000; Clone OTI1G6; OriGene), mouse monoclonal anti-Cyclin B1 (D5C10, 1:1000, Cell Signaling Technology; Danvers, MA, USA), mouse anti- proliferating cell nuclear antigen (PCNA) (PC10, 1:2000, Cell Signaling Technology), rabbit anti-Wee1 (1:1000, Cell Signaling Technology), rabbit anti-KIF11 (1:1000, Proteintech), rabbit monoclonal recombinant anti-c-Myc[Y69] (1:1000, Abcam), rabbit polyclonal anti-SAT11:1000, Proteintech) and mouse monoclonal anti-α-Tubulin (B-5-1-2, 1:2500, Sigma-Aldrich; St. Louis, MO, USA) or HRP-conjugated glyceraldehyde-3-phosphate dehydrogenase (GAPDH) (1:5000, Abcam) as control for protein loading. The secondary antibodies were HRP-conjugated swine anti-rabbit immunoglobulins and rabbit anti-mouse immunoglobulins diluted in blocking-solution (1:2500, Dako; Glostrup, Denmark). The membranes were washed three times with TBST between antibody incubations. Bound proteins were detected by enhanced chemiluminescence using ChemiDocTM Gel Imaging System (Bio-Rad).

### Cell stainings

Cells were cultured overnight on gelatin precoated coverslips and fixed with paraformaldehyde 4% for 10 min in room temperature. After incubation with block (PBS 5% BSA, 0.5% triton) for 30 min, coverslips covered with monoclonal mouse anti-CREM (clone 3B; Novusbio) diluted 1:100 in block, for 1–2 h, in room temperature. The coverslips were washed three times with PBS incubated with the Alexa Fluor 568 goat anti-mouse IgG (1:500, Invitrogen; Waltham, MA, USA). For visualization of actin filaments the cells were incubated with Phalloidin Alexa 488 diluted 1:500 in block for 1 h, at room temperature. The mounting medium contained DAPI for staining nuclei (ProLong® Gold Antifade Mountant with DAPI, Life Technologies; Carlsbad, CA, USA). After each staining step, the cells were washed three times with PBS. Images were taken with a Nikon Elipse Ni fluorescence microscope (Nicon; Tokio, Japan) and analysed with ImageJ v1.53f51 software (http://rsbweb.nih.gov/ij/).

### Senescence‐associated β‐galactosidase staining

After siRNA treatment, the cells were cultured overnight on gelatin precoated coverslips and fixed with paraformaldehyde 4% for 10 min at room temperature. Senescence‐associated β‐galactosidase staining was carried out overnight using the Senescence Cells Histochemical Staining kit (Sigma-Aldrich) according to the manufacturer instructions. All the samples were stained in duplicate and mounted in medium contained DAPI. The number of SA β‐galactosidase‐positive cells was counted and expressed as a percentage of DAPI positive cells. The experiments were repeated at least three times.

### Proliferation assessment

Cell proliferation was evaluated by calculating the percentage of Ki67 positive cells stained with mouse anti-Ki67 antibody (Dako) (1:200) followed by Alexa Fluor 568 goat anti-mouse IgG (1:500, Invitrogen). Images were taken with a Nikon Elipse Ni fluorescence microscope and analyzed with ImageJ v1.53f51 software. The number of Ki67-positive cells was counted and expressed as a percentage of DAPI positive cells. The experiment was done at least three times.

### Wound healing migration assay

After 48 h of siRNA treatment, confluent cells (40 × 10^3^) were grown overnight in 96-well Essen BioScience ImageLock microplates (Essen Bioscience; Ann Arbor, MI, USA) precoated with geltrex (Gibco). Wounds were precisely made by the 96-pin Wound-Maker provided with the IncuCyte S3 (Essen Bioscience). After washing once with PBS to remove the detached cells, 100 μL of complete medium was added and the cells were placed in the IncuCyte S3. The wound images were automatically acquired from the incubator at 2 h intervals for up to 72 h, depending on the cell line. The kinetics of the wound closure was analyzed by IncuCyte software (Essen Bioscience). The experiment was repeated four times.

### Transcriptome analysis

To investigate the changes caused by EWSR1::CREM knockdown in CHL-1 cells, we carried out a transcriptome analysis (RNA sequencing) of samples from triplicate experiments. Cells (500 × 10^3^) were plated on twelve-well plates, treated with Control or CREM siRNA and, after 48 h, RNA samples were collected and purified using the NucleoSpin RNA/Protein Kit (Macherey-Nagel; Allentown, PA, USA) according to the manufacturer's protocol. A total of 100 ng/per sample was sent for sequencing in the Turku Centre of Biotechnology sequencing core, Turku, Finland, using the Illumina HiSeq 2500 sequencing (Illumina; San Diego, CA, USA).

Results were normalized according to GAPDH expression and mean gene expression levels are reported as reads per kilobase per million (RPKM) values. For the purposes of further analysis, differentially expressed genes were subjected to a cut off of false discovery rate (FDR) < 0.05 and transcripts with Log2 Fold Change (FC) ≥ 2 or < 2 and *P* ≤ 0.05 were considered as significantly upregulated or downregulated. The volcano plot visualizing the transcriptome analysis results and cut off choices was created using the publicly available online application The VolcaNoseR: https://huygens.science.uva.nl/VolcaNoseR/^7^.

### Functional analysis and target gene prediction

Functional analysis from the Gene Ontology (GO) database^8^ was performed using the MetaScape tool publicly available at https://metascape.org^9^. Heat maps with the genes expressed in the enriched pathways were created using the publicly available online program HeatMapper: http://heatmapper.ca^10^. The list of genes of the CREM transcription factor predicted using known transcription factor binding site motifs was obtained from the TRANSFAC Predicted Transcription Factor Targets dataset https://maayanlab.cloud/Harmonizome/gene_set/CREM/TRANSFAC+Predicted+Transcription+Factor+Targets^11^ (accessed Aug. 2021). Data regarding the druggability of proteins encoded by the predicted target genes was gathered from The Drug Gene Interaction database which is publicly available online https://dgidb.org/^12^.

### Cell cycle analysis

48 h after siRNA treatment the cells were incubated and processed with a Click-iT™ EdU Pacific Blue™ Flow Cytometry assay Kits (Invitrogen) according to the manufacturer’s protocol. Flow cytometry analysis was carried out using BD LSR Fortessa™ (BD Biosciences; Franklin Lakes, NJ, USA) and data were analyzed using Flowing software 2.5.1 (Mr. Perttu Terho, Turku Bioscience Centre, Turku, Finland) to generate percentages of cells in G0/G1, S, and G2/M phases. The experiment was repeated three times.

### Total polyamines

The cellular polyamine content was evaluated 48 h after siRNA treatments using Total Polyamine Assay Kit (Fluorometric; Sigma-Aldrich). Briefly, 250 × 10^3^ pelleted cells supended in 100 µl of Polyamine Assay buffer and homogenized by passing five times through a 31-gauge needle using an insulin syringe. The samples were centrifuged at 10,000 × *g* for 5 min at 4 °C, supernatants were transferred to new tubes and incubated with 2 µl of Sample Clean-up Mix for 30 min at room temperature. After centrifuged once more at 10,000 × *g* for 5 min at 4 °C, 20 µl of the supernatants were used for measurements according to the manufacture instructions. The assay was repeated three times. Results are shown in pmol/50 × 10^3^ cells.

### Statistical analysis

Statistical analyses were performed using Excel (mRNA seq), and VassarStats website, freely available online at http://vassarstats.net/. Differences in mRNA expression, migration, cell cycle phase, Senescent or Ki67 positive percentages between control and CREM siRNA treated cells were investigated using *t*-test. *P* values less than 0.05 were considered statistically significant.

## Results

### Knockdown of EWSR1::CREM and CREM expression in CHL-1 cell line induced senescence and reduced proliferation and migration

Loss of EWSR1::CREM fusion protein in CHL-1 has previously been found to be associated with reduced proliferation, senescence and impaired invasion^3^. To validate these findings, we investigated the cellular properties associated with endogenous expression of EWSR1::CREM in this cell line. For this, we studied the effect of EWSR1::CREM knockdown on cellular properties.

**Figure 2:**
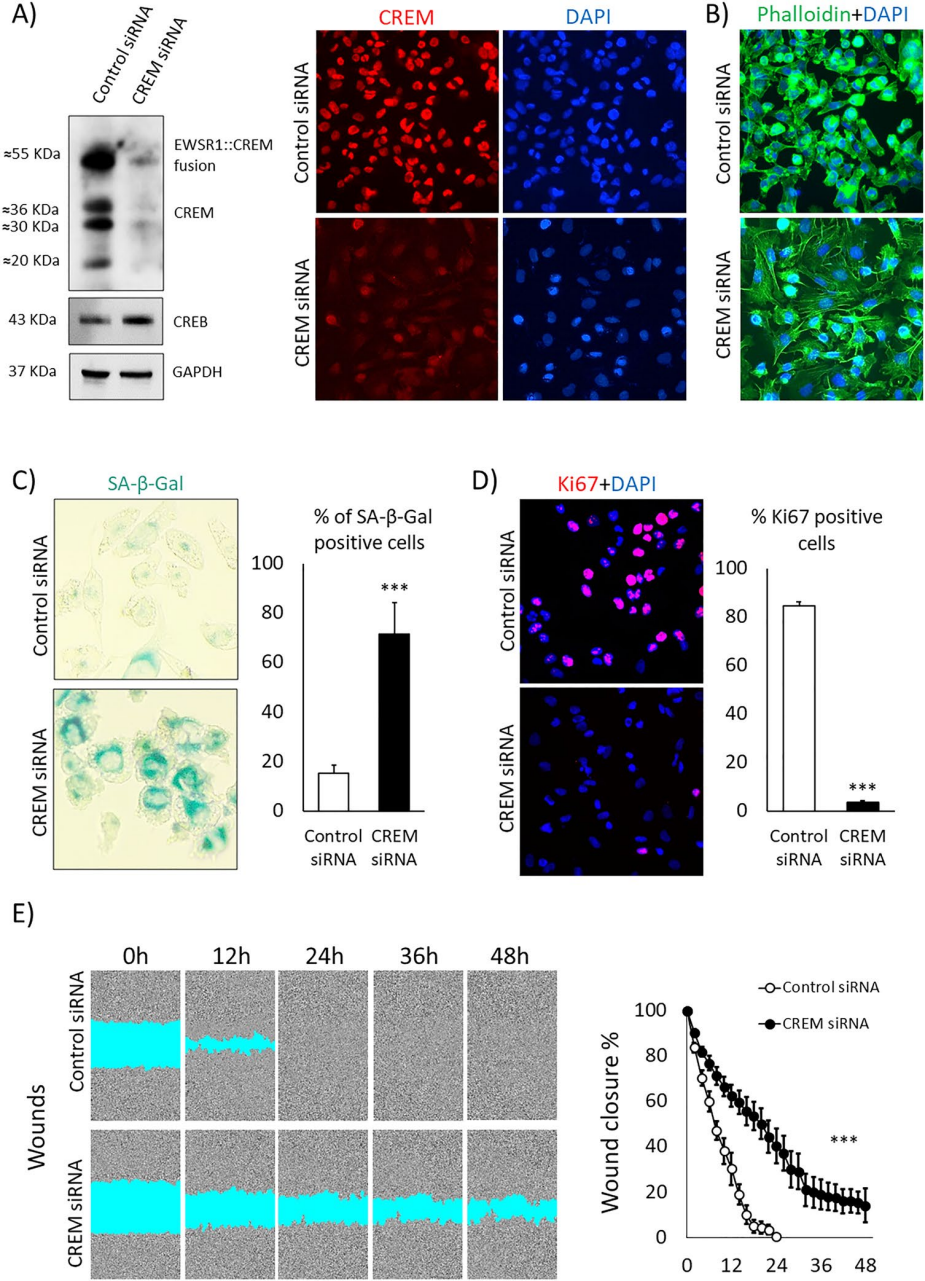
Effects of CREM knockdown in the CHL-1 cell line. (**A**) Western blotting and cell staining after CREM siRNA treatment. The blots are cropped and original blots can be found in Supplementary information—Original blots Supplementary Figs. S2 and S3. (**B**) Cell morphology after CREM knockdown. (**C**) Senescence assessed with SA β‐galactosidase staining after CREM knockdown, confirming loss of proliferation from 84 to 4%. (**D**) Immunostaining for the proliferation marker Ki67 after CREM siRNA treatment. (**E**) Wound healing assay after CREM knockdown. ***P ≤ 0.001 (*t*-test).

As we have earlier shown, the EWSR1::CREM fusion protein in CHL-1 can be detected as a band of ≈ 55 kDa size^13^. Western blotting showed that the EWSR1::CREM fusion protein was highly expressed, with a stronger band than those representing CREM isoforms. Using a pool of siRNAs targeting the DNA-binding domain of CREM, a striking reduction of EWSR1::CREM and CREM expression was seen in western blotting and cell staining (Fig. 2A). The expression of CREB was not altered. In line with EWSR1::CREM and CREM protein loss, reduction of respective mRNA expression was seen using RT-PCR using fusion specific and CREM primers (data not shown). After knockdown, cell shape shifted from spindle-like to a more enlarged and irregular shape, characteristic of cellular senescence^14^ (Fig. 2B).

Next, we measured SA β‐galactosidase positivity, which is a biomarker for senescence, and performed Ki67 staining to measure proliferation. In the CREM knockdown samples, SA β‐galactosidase positivity was substantially increased, indicating senescence (Fig. 2C) and the number of Ki67 positive cells was synchronously reduced (Fig. 2D). Migration was tested by wound healing assay, a significant delay in wound closure was seen after CREM knockdown (Fig. 2E).

### mRNA sequencing analysis identified cell cycle/mitosis pathways regulated by ESWR1-CREM and CREM in CHL-1 cells

Ideally, the effects of endogenous EWSR1::CREM protein would be studied in a cancer cell line that does not express CREM. However, CREM is ubiquitously expressed, and no such cell line has to our knowledge been discovered. Therefore, we went on to identify genes and pathways primarily regulated by the highly expressed EWSR1::CREM fusion protein in CHL-1 cells considering that a minority of the transcriptional alteration is likely attributable to the wildtype CREM protein. We performed mRNA sequencing to compare the transcriptomes of ESWR1::CREM and CREM silenced CHL-1 cells with control siRNA treated CHL-1 cells. As a result, we found that as many as nearly 14 000 genes were differentially expressed after EWSR1::CREM and CREM knockdown (data available at Gene Expression Omnibus repository, accession number GSE210911).

**Figure 3:**
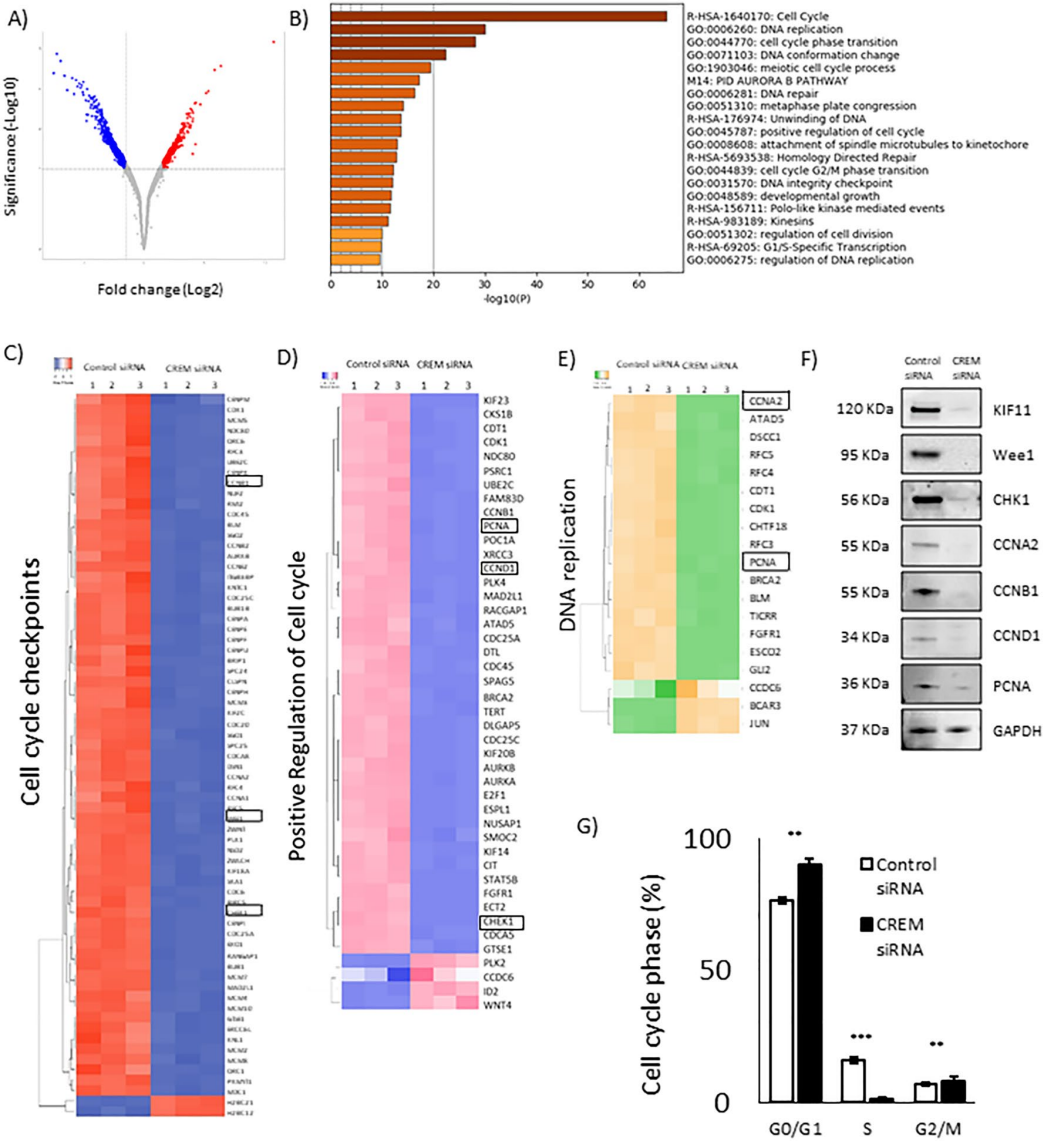
mRNA sequencing of CHL-1 cells treated with control and CREM siRNA. (**A**) Volcano plot for visualization of data selection: red for selected upregulated genes, blue for selected downregulated genes and grey for omitted genes based on significance and fold change. (**B**) Top 20 gene clusters enriched by CREM knockdown. Heatmaps of differentially expressed genes ≥ twofold change upon CREM Knockdown via CREM siRNA, with most being downregulated (upper rows) and small portion of upregulated genes (bottom rows) for (**C**) Cell cycle check points, (**D**) Positive Regulation of Cell cycle and (**E**) DNA replication. (**F**) Cell cycle markers presented in the heatmaps were investigated protein level in western blotting, GAPDH was used as protein loading. The blots are cropped and original blots/replicates can be found in Supplementary information—Original blots Supplementary Figs. S2, S4, S5, S6, S7. (**G**) Cell cycle analysis results presented by cell phases. **P ≤ 0.01 and ***P ≤ 0.001 (*t*-test).

To filter the gene list, we adopted criteria based on significant difference of *P* ≤ 0.05 and Log2 fold-change ≥ 2. With this scheme, we identified 712 genes for functional analysis. This selection of data is visualized by a volcano plot, Fig. 3A.

To analyze pathways altered, we performed a Metascape functional analysis of these 712 genes. This analysis revealed that ESWR1-CREM and CREM knockdown altered pathways involved in cell cycle, DNA replication and cell cycle phase transition and other pathways (Fig. 3B).

Heatmaps of genes in representative pathways of cell cycle checkpoints, positive regulation of cell cycle, and DNA replication are shown in Fig. 3C,D and E. The vast majority of the genes were downregulated in these three pathways after EWSR1::CREM and CREM knockdown.

Next, we wanted to validate identified downstream genes at protein level using immunoblotting. We chose the following genes: two G2 checkpoint markers; WEE1 G2 checkpoint kinase (WEE1) and Cyclin B1 (CCNB1), G1 checkpoint marker Cyclin D1 (CCND1), G1 and G2 checkpoint marker: Cyclin A2, (CCNA2), Checkpoint kinase 1 (CHK1) that is responsible for checkpoint mediated cell cycle arrest in response to DNA damage or the presence of unreplicated DNA, representing one of the kinesins—Kinesin family member 11 (KIF11) and proliferation marker Proliferating cell nuclear antigen (PCNA). The results confirmed the reduction of all of the chosen gene products further validating our results. (Fig. 3F).

As cell cycle was the main process affected by EWSR1::CREM and CREM knockdown in CHL-1, we conducted cell cycle analysis to investigate the phase that would be altered. In this analysis, we found that the proportions of cells in S and G2/M phases were reduced after the knockdown of EWSR1::CREM and CREM, while the proportion of cells in the G0/G1 phase increased from 77 to 91% as cells were not able to progress from G1 or became senescent (Fig. 3G). FACS analysis showed that CREM knockdown inhibits S phase progression in CHL-1 cells.

### Wild type CREM knockdown reduced proliferation in HEK293, PC-3, and WM164 cells

The cell cycle was the main cellular process affected by EWSR1::CREM and CREM knockdown in CHL-1 cells. We wondered if CREM knockdown in cell lines lacking the constitutionally active fusion protein would result in a similar effect. To interrogate the effect of CREM knockdown on non-malignant and malignant cells, we used three cell lines: Human embryonic kidney cell line HEK 293, human prostate carcinoma cell line PC-3, and human melanoma WM164 cell line.

**Figure 4:**
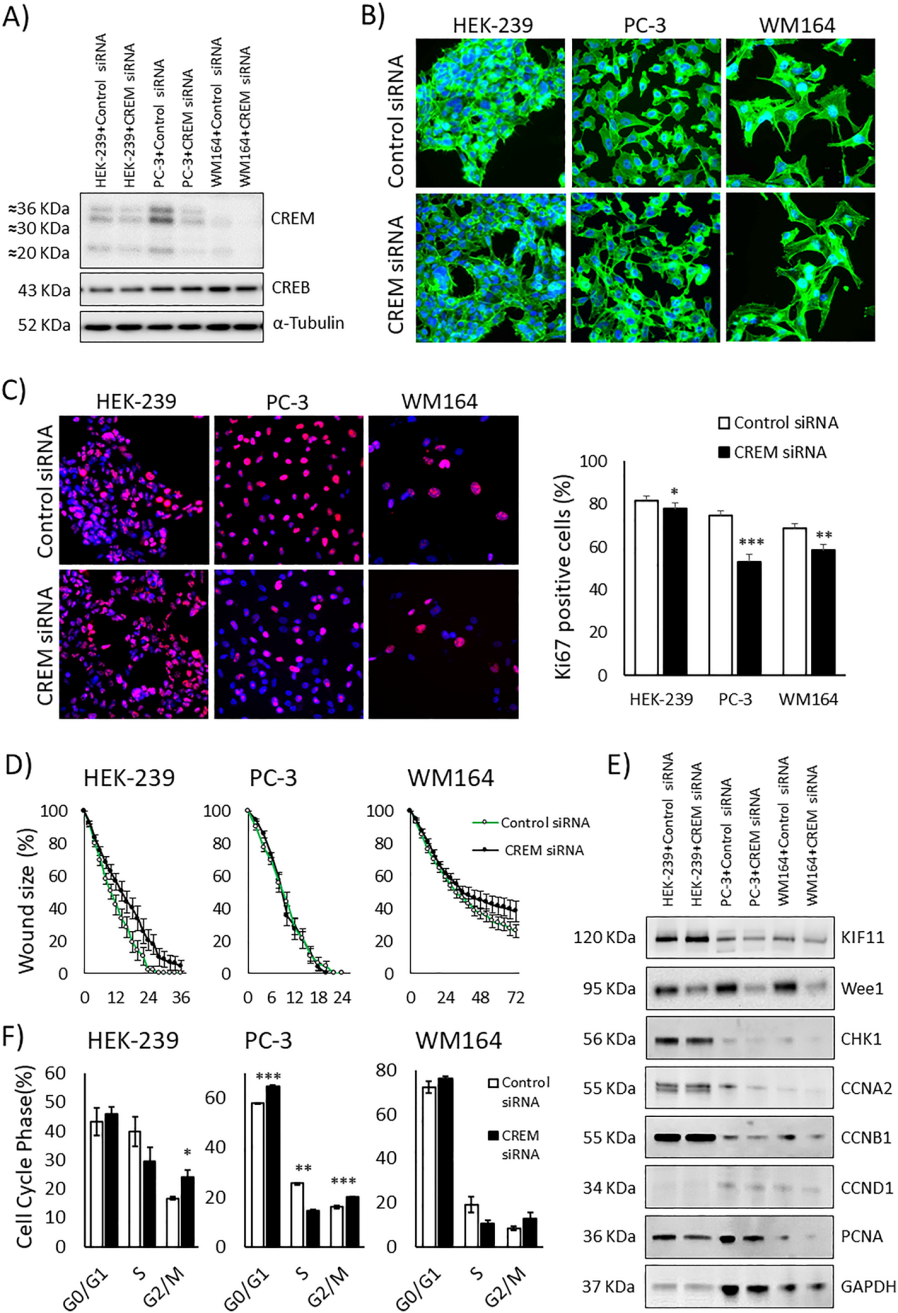
Wild type CREM knockdown. (**A**) Immunoblot showing Wild type CREM knocked down in HEK-293, PC3, and WM164 after treatment with CREM siRNA for 48 h. The blots are cropped and original blots can be found in Supplementary information—Original blots Supplementary Fig. S8. (**B**) Cell morphology in HEK-293, PC3, and WM164 cell lines after the reduction in CREM levels. (**C**) Proliferation assessed with Ki67 after CREM siRNA in HEK-293, PC3, and WM164 cell lines, it resulted with proliferation of loss from 82 to 78%, from 75 to 53%, and from 69 to 58%, respectively. (**D**) Wound healing assay to assess migration after CREM knockdown in HEK-293, PC3, and WM164 cell lines. (**E**) Downstream cell cycle related proteins and their expression measured by immunoblotting after CREM knockdown in HEK-293, PC3, and WM164 cell lines. The blots are cropped and CCNA2 was run in lanes with one empty in between so for this image, the empty lane is removed. Original blots/replicates can be found in Supplementary information—Original blots Supplementary Figs. S2, S4, S5, S6 and S7. (**F**) FACS analysis results after CREM siRNA treatment for HEK-293, PC3, and WM164 cell lines. *P ≤ 0.05, **P ≤ 0.01, and ***P ≤ 0.001 (*t*-test).

Cells were transfected with CREM siRNA for 48 h. Reduced expression of CREM protein was confirmed by immunoblotting (Fig. 4A). Bands corresponding to CREM predicted size were clearly weaker after knockdown, with most efficient loss of CREM seen in PC-3 and WM164 cells. Morphologically, no significant alterations could be observed (Fig. 4B). Neither was a significant change observed in the frequency of senescent cells (data not shown). Proliferation was assessed using Ki67 staining and quantification of positively stained nuclei. The knockdown of CREM resulted in a modest reduction in proliferation in HEK-293, PC-3, and WM164 (Fig. 4C). In the wound healing assay, a slight delay in migration was observed solely in HEK-293 cells after CREM knockdown (Fig. 4D). No effect was observed in PC-3 or WM164 cells.

In order to investigate if the downstream cell cycle related proteins were altered as seen in the CHL-1 cells, immunoblotting for Wee1, CCNB1, CCND1, CCNA2, CHK1, KIF11 and PCNA was conducted. HEK-293 showed reduction of Wee1 expression. PC-3 showed reduction of Wee1 and CCNA2 expression. WM164 showed reduction of Wee1, CCNB1, CCNA2 and PCNA expression (Fig. 4E). The result reflects the lesser impact of CREM knockdown in these non-fusion bearing cell lines.

To analyze the effects of CREM knockdown on the cell cycle, we conducted FACS analysis. Here, results showed a reduction of cells in S phase after CREM siRNA treatment in HEK-293, PC-3, and WM164 cells (Fig. 4F). Only PC-3 cells demonstrated significant changes in all cell phase groups although all cell types had a similar effect as seen in CHL-1, but of smaller magnitude.

### CREM target genes with altered expression after EWSR1::CREM and CREM loss in CHL-1 cells

Taken together, the results above indicate that the best part of alterations seen upon CREM knockdown in the CHL-1 cell line are attributable to the highly expressed fusion protein EWSR1::CREM, which by structure is predicted to enhance transcription from CREM target genes. The effects of CREM knockdown on cell lines lacking this fusion were, as expected, similar but of lesser amplitude.

The loss of EWSR1::CREM and CREM expression lead to a dramatic loss of proliferation and induction of senescence in CHL-1, but it remained unclear whether this was due to differential expression from direct CREM target genes, or alterations further downstream. In pursuance of direct CREM target genes responsible for the altered phenotype, we reassessed the differentially expressed gene list from our transcriptomic analysis, looking for direct CREM target genes. To identify CREM regulated genes, we used the TRANSFAC Predicted Transcription Factor Targets dataset that can be utilized to identify potential transcription factor binding sites. We found 660 predicted CREM target genes. Among these, 497 genes had been differentially expressed in our transcriptomic analysis of CHL-1 cells with decrease of EWSR1::CREM and CREM expression. From these 497 genes, 248 were significantly changed (*P* ≤ 0.05) (128 downregulated and 120 upregulated), when all fold-changes were considered. Adopting a fold-change cut off of Log2 ≥ 2, we found 23 genes; 18 downregulated and 5 upregulated.

Since the EWSR1::CREM fusion protein is a constitutively active transcription factor, the genes downregulated by EWSR1::CREM and CREM knockdown were of greatest interest. In addition, we listed upregulated genes, since specific isoforms of wild type CREM can repress expression of CREM target genes.

The majority of downregulated CREM target genes are involved in cell cycle related Gene Ontology biological processes according to the freely accessible UniProt website www.uniprot.org/^15^. Eleven out of the 23 predicted target genes are druggable according to The Drug Gene Interaction Database. The predicted CREM target genes differentially expressed upon EWSR1::CREM and CREM knockdown, their biological process and potential druggability are listed in Table 1.

**Table 1:** Predicted CREM target genes that were significantly changed with a fold change of Log2 ≥ 2 after CREM siRNA treatment in the CHL-1 cell line.

Gene	Name	*P*-value	log2FC	RPKM normalized (GAPDH)	Gene ontology—biological process	Druggability
Downregulated						
DACT2	Dishevelled binding antagonist of beta catenin 2	0.0001	− 5.926	299	Multiple, including epithelial cell morphogenesis, negative regulation of cell adhesion and negative regulation of nodal signaling pathway	
ODC1	Ornithine decarboxylase 1	0.0005	− 3.536	3185	Multiple, including polyamine biosynthesis, putrescine biosynthetic process from ornithine and regulation of protein catabolic process	Druggable
KIF11	Kinesin family member 11	0.002	− 3.322	95	Multiple, including cell division, mitotic cell cycle and mitotic centrosome separation	Druggable
SKA1	Spindle and kinetochore associated complex subunit 1	0.001	− 2.886	31	Multiple, including cell division, chromosome segregation and mitotic cell cycle	
SAT1	Spermidine/spermine N1-acetyltransferase 1	0.002	− 2.721	36	Multiple, including putrescine catabolic process, polyamine biosynthetic process and angiogenesis	
VEPH1	Ventricular zone expressed PH domain containing 1	0.002	− 2.701	< 1	Regulation of signal transduction, negative regulation of transforming growth factor beta receptor signaling pathway and negative regulation of SMAD protein signal transduction	
APCDD1	APC down-regulated 1	0.003	− 2.531	6	Hair follicle development and negative regulation of Wnt signaling pathway	
CALCB	Calcitonin related polypeptide beta	0.003	− 2.503	5	Multiple, including G protein-coupled receptor signaling pathway, negative regulation of inflammatory response to antigenic stimulus and cellular calcium ion homeostasis	Druggable
LPAR3	Lysophosphatidic acid receptor 3	0.003	− 2.307	< 1	Multiple, including activation of MAPK activity, positive regulation of collateral sprouting, and chemical synaptic transmission	Druggable
CAPN3	Calpain 3	0.003	− 2.279	26	Multiple, including G1–G0 transition involved in cell differentiation, negative regulation of apoptotic process and negative regulation of transcription, DNA-templated	Druggable
LYST	Lysosomal trafficking regulator	0.004	− 2.261	12	Multiple, including melanosome organization, phagocytosis and protein transport	
RRM1	Ribonucleotide reductase catalytic subunit M1	0.003	− 2.257	177	Multiple, including DNA replication, mitotic cell cycle and cell proliferation in forebrain	Druggable
PEG3	Paternally expressed 3	0.003	− 2.254	165	Multiple, including regulation of gene expression, regulation of transcription by RNA polymerase II and apoptotic process	
B3GALT2	Beta-1,3-galactosyltransferase 2	0.005	− 2.2	1	Multiple, including protein glycosylation, galactosylceramide biosynthetic process and oligosaccharide biosynthetic process	
C4orf46	Chromosome 4 open reading frame 46 (former RCDG1)	0.004	− 2.125	27	N/A	
ITGB3BP	Integrin subunit beta 3 binding protein	0.004	− 2.115	25	Multiple, including cell division, apoptotic process and regulation of transcription, DNA-templated	
RTN4R	Reticulon 4 receptor (former NOGOR)	0.004	− 2.068	9	Multiple, including positive regulation of Rho protein signal transduction, neuronal signal transduction and negative regulation of axonogenesis	Druggable
CEP78	Centrosomal protein 78	0.004	− 2.018	72	Multiple, including G2/M transition of mitotic cell cycle, ciliary basal body-plasma membrane docking and cilium organization	
Upregulated						
LRRN1	Leucine-rich repeat neuronal protein 1	0.0002	4.197	1954	Positive regulation of synapse assembly	Druggable
JUN	Jun proto-oncogene, AP-1 transcription factor subunit	0.002	3.043	18	Multiple, including regulation of cell cycle, regulation of cell population proliferation and angiogenesis	Druggable, Clinically actionable
UBE2T	Ubiquitin conjugating enzyme E2 T	0.003	2.444	57	Multiple, including DNA repair, cellular response to DNA damage stimulus and protein ubiquitination	Clinically actionable
PTP4A1	Protein tyrosine phosphatase 4A1	0.003	2.441	374	Multiple, including cell cycle, multicellular organism development and positive regulation of cell migration	Druggable
LRRC8C	Leucine rich repeat containing 8 VRAC subunit C	0.003	2.396	33	Multiple, including transmembrane transport, protein hexamerization and cellular response to osmotic stress	

**Figure 5:**
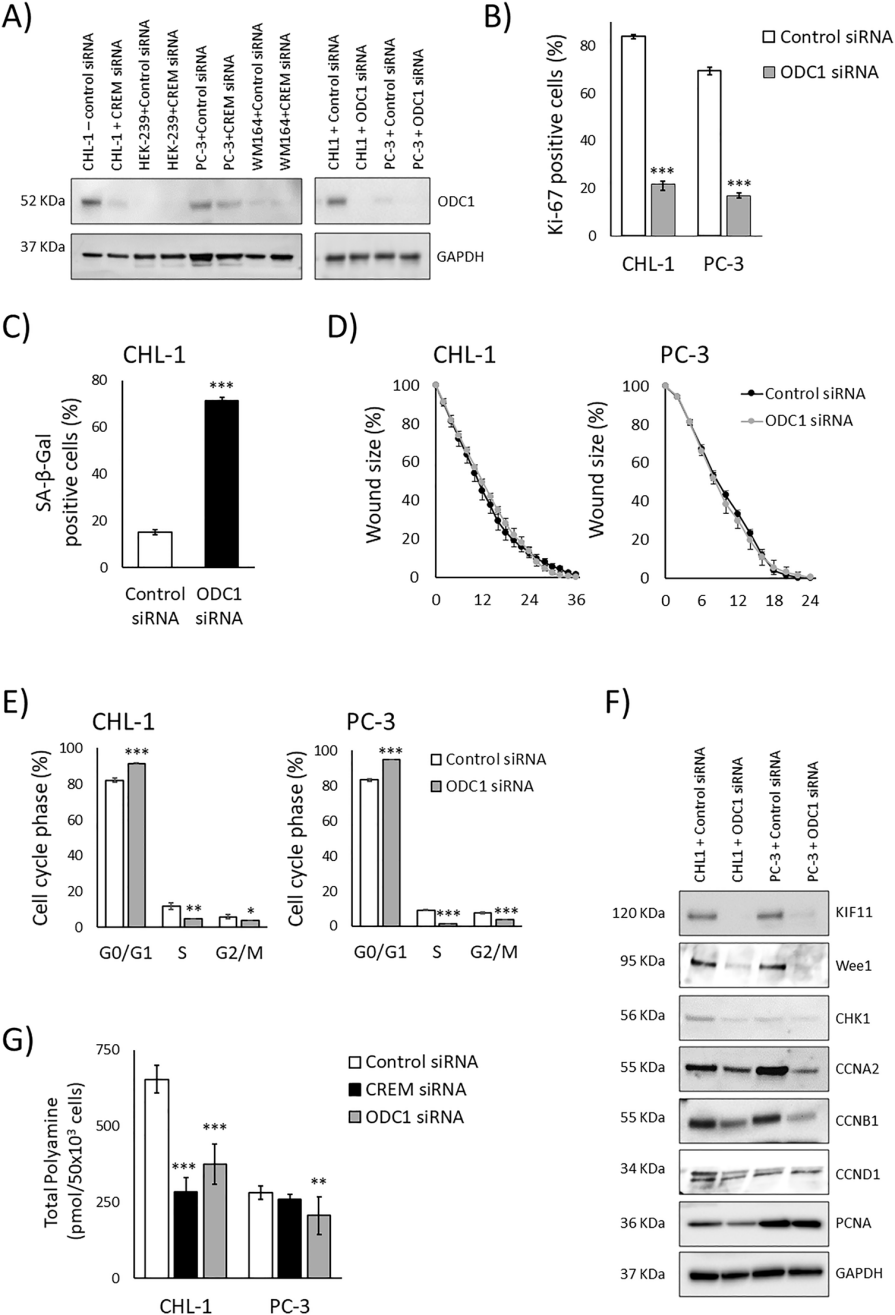
Effects of CREM and ODC1 silencing on cell lines CHL-1 and PC-3. (**A**) Immunoblot showing effects of CREM knockdown and ODC1 knockdown on ODC1 protein levels in in CHL-1, HEK-293, PC3, and WM164 cell lines. The blots are cropped and original blots can be found in Supplementary information—Original blots Supplementary Figs. S9 and S10. (**B**) Proliferation assessed with Ki67 after ODC1 siRNA in CHL-1 and PC3 cell lines. Loss of proliferation was 84–22% in CHL-1 and from 69 to 17% in PC3 cells. (**C**) Senescence assessed with SA β‐galactosidase staining after ODC1 knockdown in CHL-1 cell line, an increase from 15 to 71% was seen. (**D**) Wound healing assay to assess migration after ODC1 knockdown in CHL-1 and PC3 cell lines. (**E**) FACS analysis results after ODC1 siRNA treatment for CHL-1 and PC3 cell lines. An increase in G0/G1 in CHL-1cells from 82 to 91% and PC3 in cells from 83 to 95% (**F**) Downstream cell cycle related proteins and their expression measured by immunoblotting after ODC1 knockdown in CHL-1 and PC3 cell lines. The blots are cropped and original blots/replicates can be found in Supplementary information—Original blots Supplementary Figs. S11, S12 and S13. (**G**) Total polyamine concentration compared in CHL-1 and PC3 cell lines after CREM and ODC1 knockdown. *P ≤ 0.05, **P ≤ 0.01, and ***P ≤ 0.001 (*t*-test).

Among the CREM target genes downregulated upon EWSR1::CREM and CREM knockdown, the highest relative mRNA expression before knockdown was noted for Ornithine decarboxylase 1 (ODC1). ODC1 is a well characterized enzyme responsible of decarboxylation of ornithine into putrescine^16^. This is the first phase of polyamine biosynthesis, a rate-limiting step that regulates cell proliferation^17,18^. Using western blotting, we established that the reduction of ODC1 mRNA is translated to loss of ODC1 protein (Fig. 5A). Another CREM target gene with reduced expression upon EWSR1::CREM and CREM knockdown also regulates polyamines: Spermidine/spermine N1-acetyltransferase 1 (SAT1, also known as SSAT). SAT1 is a catabolic enzyme that catalyzes the acetylation of two of the three polyamines: spermidine and spermine, leading to the degradation or transport of these polyamines out of cells^19^.The relative mRNA expression of SAT1 before knockdown was considerably lower than that of ODC1. Nevertheless, we were able to validate that the reduction of SAT1 mRNA also translates to reduced protein expression using western blotting (see Supplementary Fig. S1 online).

The extent of ODC1 loss, together with its known role in proliferation, suggested it may be the pivotal mediator of the cellular effects brought about by upon EWSR1::CREM and CREM knockdown.

ODC1 expression is regulated by the MYC proto-oncogene, bHLH transcription factor (MYC)^20^.To rule out a secondary ODC1 loss, through MYC, we checked the effect of upon EWSR1::CREM and CREM knockdown on MYC expression. In the transcriptomic analysis, MYC expression was unaltered. We further performed western blotting, where a slight reduction of c-myc protein expression was seen (see Supplementary Fig. S1 online).

### ODC1 is essential for cell cycle progression in the fusion bearing CHL-1 cell line

We decided to focus on the exploration and validation of the newly discovered CREM-ODC1 axis. Our hypothesis was that ODC1-mediated polyamine synthesis has a pivotal role in mediating the EWSR1::CREM and CREM effects on cell cycle and migration.

First, we used western blot to study the possible reduction of ODC1 protein expression after CREM knockdown in the fusion bearing and control cell lines. The reduction is striking in the CHL-1 cell line. With the PC-3 cell line a reduction is clear but HEK-293 or WM164 cell lines have no detectable reduction of ODC1 expression (Fig. 5A). Based on these findings we decided to continue the experiments with CHL-1, using PC-3 as the control cell line.

Next, we tested whether ODC1 silencing could induce similar functional effects as detected upon CREM silencing. Using a pool of siRNAs targeting ODC1, a dramatic reduction of ODC1 expression was seen in western blotting and cell staining in the CHL-1 cell line. In the PC-3 cell line, ODC1 expression was initially low, and further reduced by knockdown (Fig. 5A). We measured SA β‐galactosidase positivity to assess senescence and performed Ki67 staining and quantification of positively stained nuclei to measure proliferation. The number of Ki67 positive cells was reduced in both CHL-1 and PC-3 cells, confirming decrease of proliferation (Fig. 5B). The ODC1 knockdown of CHL-1 cells substantially increased SA β‐galactosidase positivity indicating increased senescence (Fig. 5C). PC-3 cells showed no significant increase in SA β‐galactosidase positivity (data not shown). Migration was tested by wound healing assay. Interestingly, no significant change was noted in either cell line (Fig. 5D).

To analyze the effects of ODC1 knockdown on the cell cycle, we conducted FACS analysis. Here, results showed in both cell lines significant reduction of cells in S and G2/M phases after ODC1 siRNA treatment. Significant increase in G0/G1 phases was observed in CHL-1 cell line and in PC-3 cell line (Fig. 5E). The cell cycle alterations were of similarmagnitude as with CREM knockdown.

We were then interested to see how the CREM downstream genes involved in cell cycle were affected by the ODC1 knockdown. In both cell lines we saw reduction of Wee1, CCNB1, CCNA2, CHK1 and KIF11 expression (Fig. 5F).

Finally, we measured the total polyamine concentration in both cell lines and compared the effects of CREM siRNA and ODC1 siRNA knockdown on the total polyamine concentration. In CHL-1, both CREM siRNA and ODC1 siRNA knockdown produced a significant reduction in the total polyamine concentration. In the PC-3 cell line, only ODC1 siRNA knockdown produced modest, but statistically significant reduction (Fig. 5G). This suggests that PC3 has compensatory mechanisms maintaining intracellular polyamine levels, and is not as dependent on ODC1 as CHL-1 is.

All in all, we were able to demonstrate that CREM siRNA and ODC1 siRNA knockdown lead to very similar phenotypes. This suggests that the phenotype is largely brought upon by ODC1. The ODC1 mediated effect does not seem to extend to migration during the studied timeframe in these cell lines.

## Discussion

Detection of fusion genes has been augmented by the development of next generation sequencing (NGS) methods. RNA-based NGS is able to look for a wide range of possible fusion genes and further has the advantage of being able to detect unknown fusion partners^21^. In consequence, new diagnostic/molecular entities with characteristic gene fusions are emerging. One of the recurring fusion genes in cancer is *EWSR1::CREM*, which has been detected in a wide variety of tumor types ranging from low-grade indolent to aggressive. Some of these tumours include: hyalinizing clear cell carcinoma^22^, myxoid mesechymal tumours^23^, angiomatoid fibrous histiocytoma^24^, clear cell sarcoma^25^, breast invasive ductal carcinoma^4^, astrocytoma^4^, renal clear cell carcinoma^4^, paraganglioma^26^ and melanoma^3^. Regardless of the relatively high frequency of this fusion, knowledge of its function and participation in oncogenesis is scarce, nor are there currently any targeted therapies available. Development of such therapies rely on the study of fusion-bearing cell lines.

Previously, Giacomini et al. found this fusion gene in the melanoma cell line CHL-1^3^. In cell studies, they found that the fusion bearing melanoma cells that were transfected with CREM-targeting siRNAs resulted in decreased cell proliferation and invasion as well as increased numbers of senescent cells. CHL-1 is, to our knowledge, the only available cell line that carries this fusion gene endogenously. To shed more light on the oncogenic properties of the chimeric EWSR1::CREM protein, we studied the functional and transcriptomic consequences of chimeric protein depletion in the CHL-1 cell line, using siRNA mediated knockdown. This cell line also expresses CREM, a gene with multiple isoforms expressed, capable of inducing or repressing expression from target genes. The CREM-targeting siRNA nearly eliminates not only the EWSR1::CREM fusion protein, but also the endogenous CREM from the CHL-1 cell line. In this setting, the effects of EWSR1::CREM and CREM proteins cannot be thoroughly uncoupled. In order to approximate the effect of the endogenous CREM in the fusion bearing cell line, we used benign and malignant cell lines without the fusion but with CREM expression as controls. In control cell lines, CREM loss reduced proliferation, but to a lesser extent as compared to the consequences of CREM and EWSR1::CREM protein loss in the fusion bearing cell line, As an example of the scale difference, CREM siRNA treatment in the melanoma cell line WM164 reduced proliferation from 69% to 58%, while the CREM siRNA in the fusion bearing CHL-1 cell line resulted in a 84% to 4% reduction. This supports that the highly expressed fusion protein is as a major driver behind the change in CHL-1. Moreover, the fusion protein can be predicted to have a dominating effect, based on the presence of EWSR1 transcription activation domains^27,28^.

In our functional cell studies with siRNA mediated knockdown of EWSR1::CREM and CREM in CHL-1 cells, we demonstrated a dramatic decrease in proliferation, impaired migration and an increased percentage of senescent cells, thus confirming the findings of Giacomini et al. In the transcriptomic analysis, we found that the loss of EWSR1::CREM and CREM had a fundamental effect. From a functional analysis, we were able to identify the core function of EWSR1::CREM and CREM in G1/S transition. This was further validated by findings in cell cycle analysis.

In transcriptomic analysis, we observed that the changes upon EWSR1::CREM and CREM knockdown were wide: as many as 14,000 genes had altered expression. This is not entirely unexpected, since CREM is a transcription factor with a multitude of potential target genes. The target genes, in turn, may also have transcriptional activities, creating diversity in affected genes. Therefore, we focused on direct CREM target genes with markedly altered expression. Using this strategy, we found that a known oncogenic gene, *ODC1*, was among the top hits. ODC1 is an enzyme, which through overexpression has capacity to transform cultured cells, with uncontrolled proliferation and tumor formation as consequence^29^. ODC1 catalyzes a rate-limiting step in polyamine synthesis, a reaction that produces putrescine, which is further processed to spermidine and spermine. Putrescine, spermidine and spermine are natural polyamines. Polyamines are essential for several fundamental cellular processes, such as synthesis of nucleic acid, protein synthesis and proliferation. ODC1 enhances polyamine synthesis, and keeps cell replication active even when cells are in a hypoxic and nutritionally deprived environment^18^. Among the direct target genes was also another polyamine related gene; SAT1. It is a catabolic enzyme that results in either the degradation or the exporting of spermine and spermidine. We saw a decrease in SAT1 levels after EWSR1::CREM and CREM knockdown. SAT1 mRNA expression was a hundred times lower than of ODC1 before knockdown. Nevertheless, SAT1 likely to some extent contributes to the knockdown phenotype. With CREM being a transcription factor with multiple direct target genes, it is very likely there are also indirect pathways that play a role in regulating the polyamine homeostasis.

By directly silencing ODC1 expression, we were able to show that maintaining polyamine synthesis, a proliferative phenotype and evasion of senescence in CHL-1 is similarly dependent on expression of EWSR1::CREM and ODC1. The novel CREM-ODC1 axis was seen in the prostate cancer cell line PC-3 as well, but the effect was not as strong. This may be explained by the very high expression of the EWSR1::CREM and ODC1 proteins in the CHL-1 cell line. This is, to our knowledge, the first report on ODC1 expression being regulated by CREM or EWSR1::CREM. Several studies have earlier shown that one of the most important cancer-associated transcription factors that enhances ODC1 expression is MYC^20^. In our transcriptomic analysis the CHL-1 cell line MYC expression was unaltered. However, on protein level we saw a slight reduction of c-myc. Therefore MYC may also contribute to the ODC1 downregulation, as part of the indirect consequences of EWSR1::CREM and CREM knockdown.

The association of EWSR1::CREM and ODC1 has potential clinical relevance for development of targeted treatments. Overexpression of ODC1 has been reported in many types of cancer^30,31^. In fact, ODC1 has been tested as a drug target, it can be pharmacologically inhibited by an FDA and EMA approved inhibitor; difluoromethylornithine (DFMO). DFMO has been utilized as an ODC1 inhibitor in various cancer models, yet in clinical use it has yielded limited results. However, a recent study has shown that by combining DFMO with AMTX 1501—a polyamine transport inhibitor, a potent anti-tumor effect was seen in vitro and in a mouse model of the malignant pediatric brain tumor Diffuse pontine glioma^32^. As it happens, a clinical trial is bring currently conducted with the combination of DMFO and AMTX 1501 (NCT03536728). Further studies on tumors with *EWSR1::CREM* fusion genes are needed to determine whether ODC1 overexpression is a general feature.

In conclusion, our results from the melanoma cell line CHL-1 confirm that EWSR1::CREM is an oncogenic fusion protein with cell cycle accelerating properties leading to increased proliferation, increased migration and evasion of senescence. The oncogenic properties of the fusion seem to be largely mediated through the altered expression of ODC1. Further research is needed to explore the treatment possibilities in *EWSR1::CREM* fusion bearing tumors.

## Supplementary Information

Supplementary Information 1.

Supplementary Information 2.

## Data Availability

The datasets generated during the current study are available in the Gene Expression Omnibus (GEO) repository, accession number GSE210911. The authors declare that all other relevant data of this study are available within the article or from the corresponding author upon reasonable request.
