# Supplementary figures and images for: The oncogenic properties of the EWSR1::CREM fusion gene are associated with polyamine metabolism
Source: Sci Rep. 2023 Mar 25;13:4884. doi: 10.1038/s41598-023-31576-x (PMC10039922; doi:10.1038/s41598-023-31576-x)

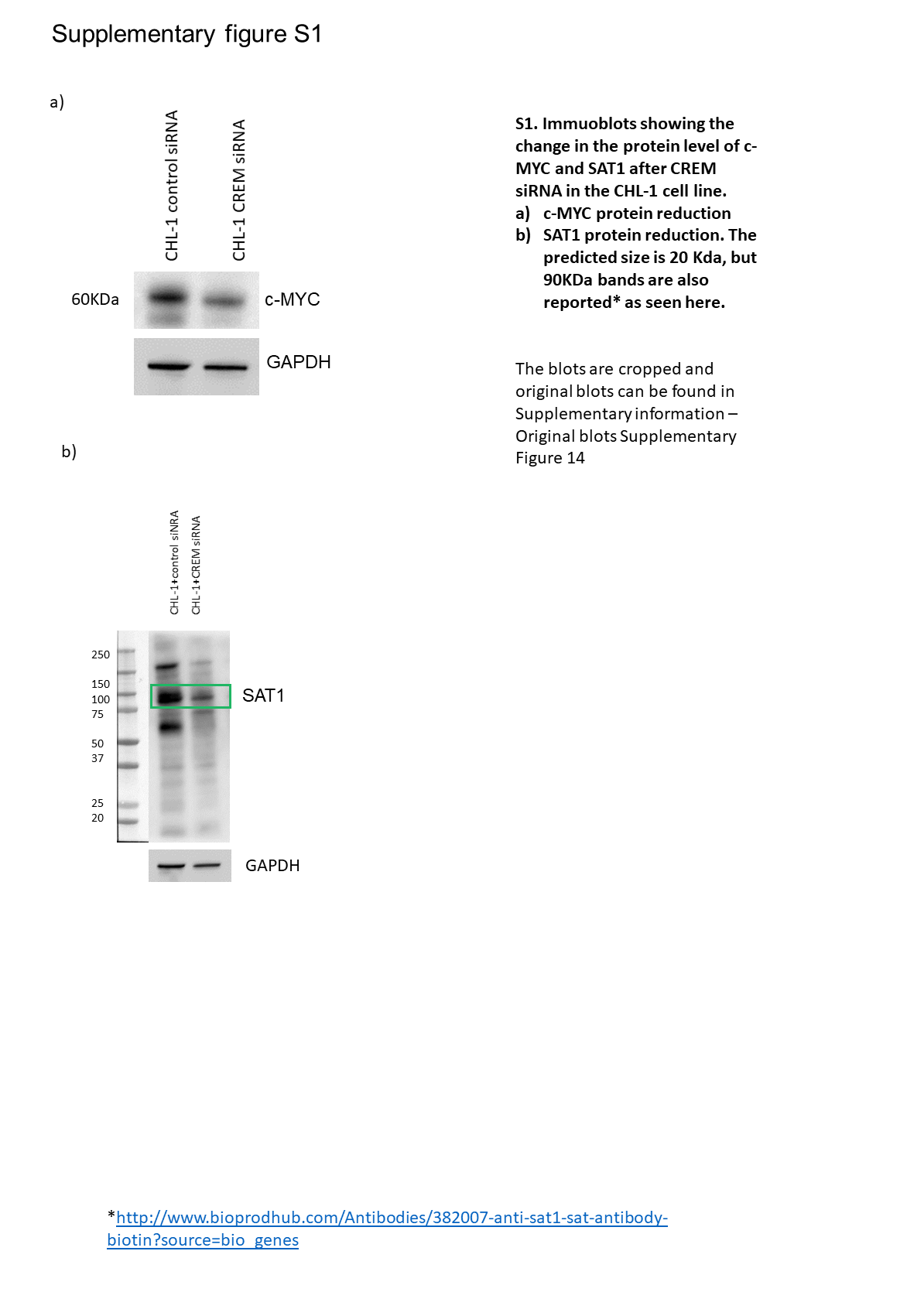

Supplement: Supplementary file 1 — Supplementary Information 1. [file 41598_2023_31576_MOESM1_ESM.png]
