# Supplementary material for: The oncogenic properties of the EWSR1::CREM fusion gene are associated with polyamine metabolism
Source: Sci Rep. 2023 Mar 25;13:4884. doi: 10.1038/s41598-023-31576-x (PMC10039922; doi:10.1038/s41598-023-31576-x)
Supplement: Supplementary file 2 — Supplementary Information 2. [file 41598_2023_31576_MOESM2_ESM.pdf]

Suplementary information - Original blots

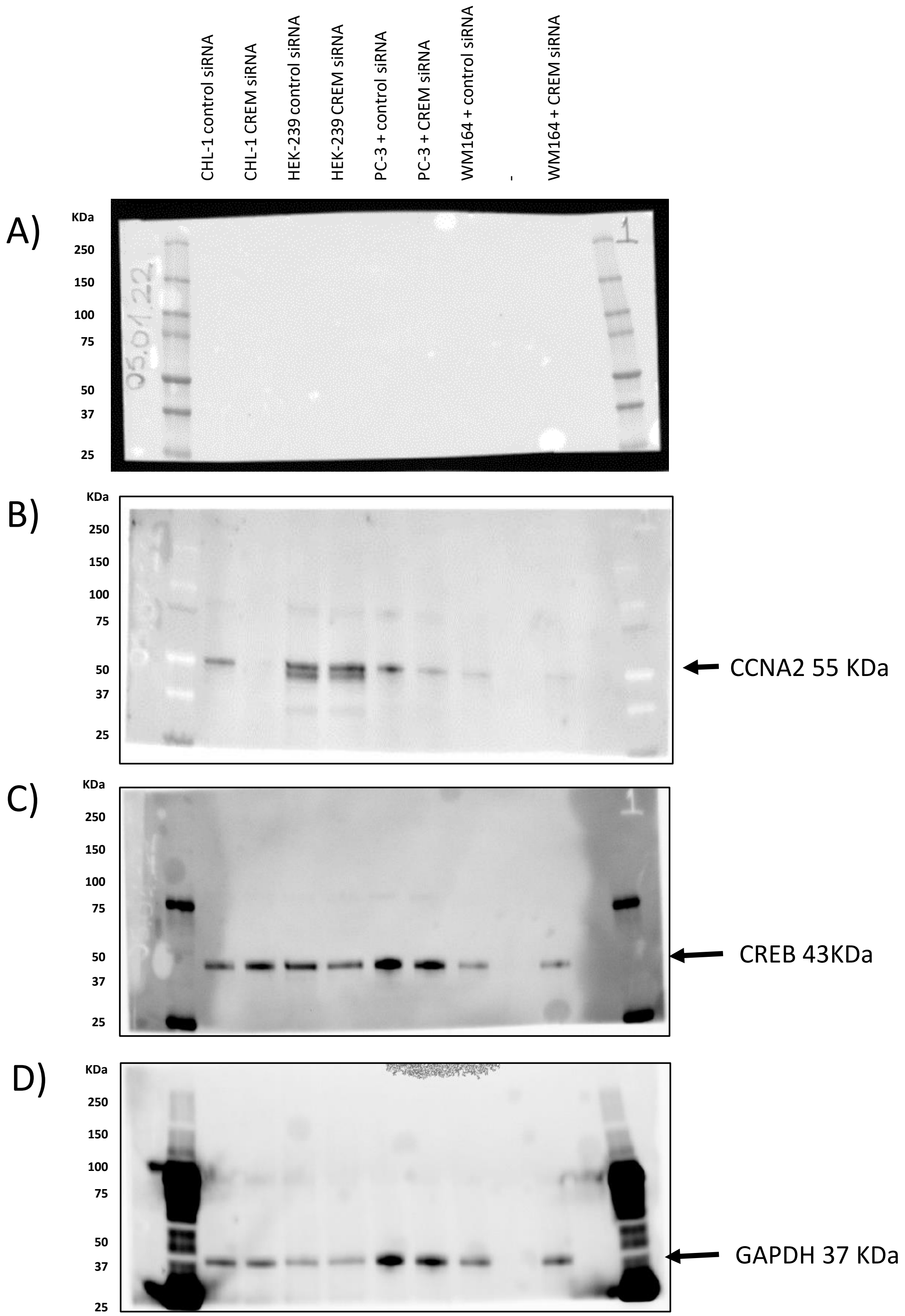

Supplementary Figure 2. The full image of western blot membrane showing the ladder (A). The membrane was stripped off, and marked with anti-CCNA2 (B; used in Figure 3F and Figure 4E), CREB (C; used in Figure 2A) , or GAPDH antibody.

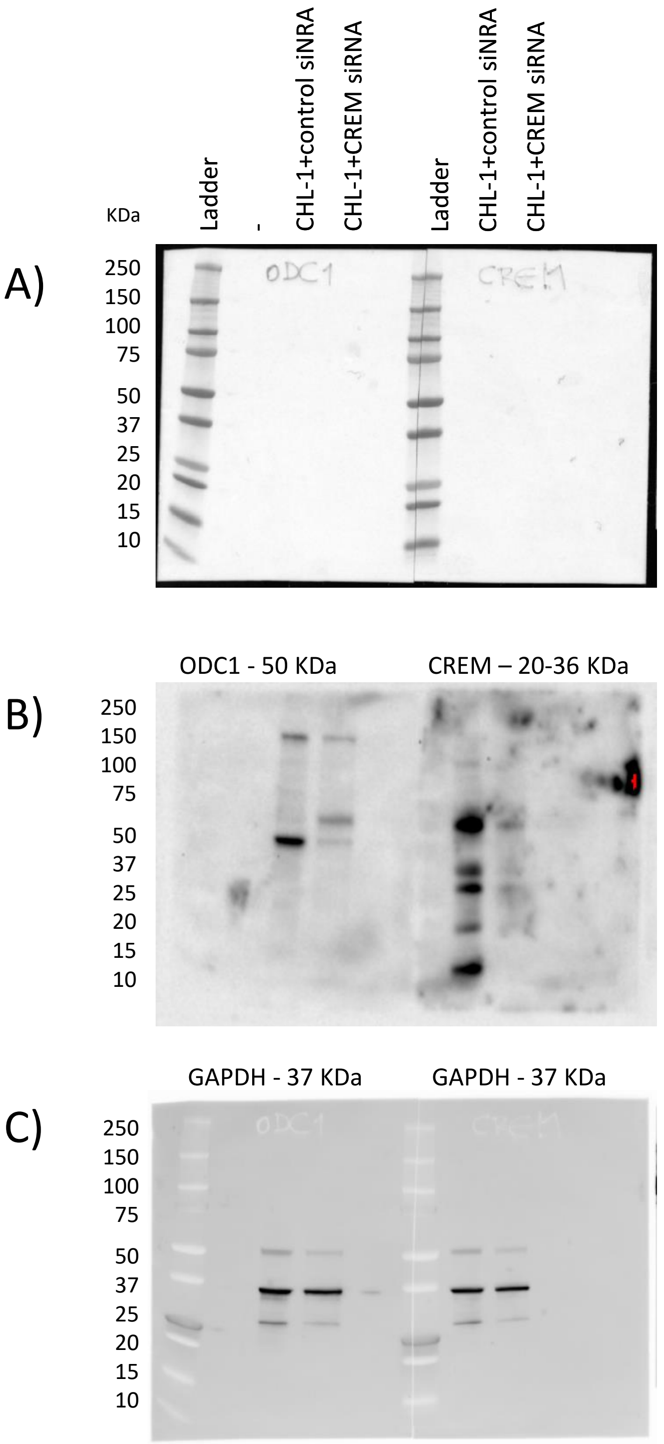

Supplementary Figure 3. The full image of membranes and western blots (A) marked with anti-ODC1 (B, left half), and CREM antibody (B, right half; used in Figure 2A). Same membrane was striped off and reprobed using anti-GAPHD antibody (C; used in Figure 2A).

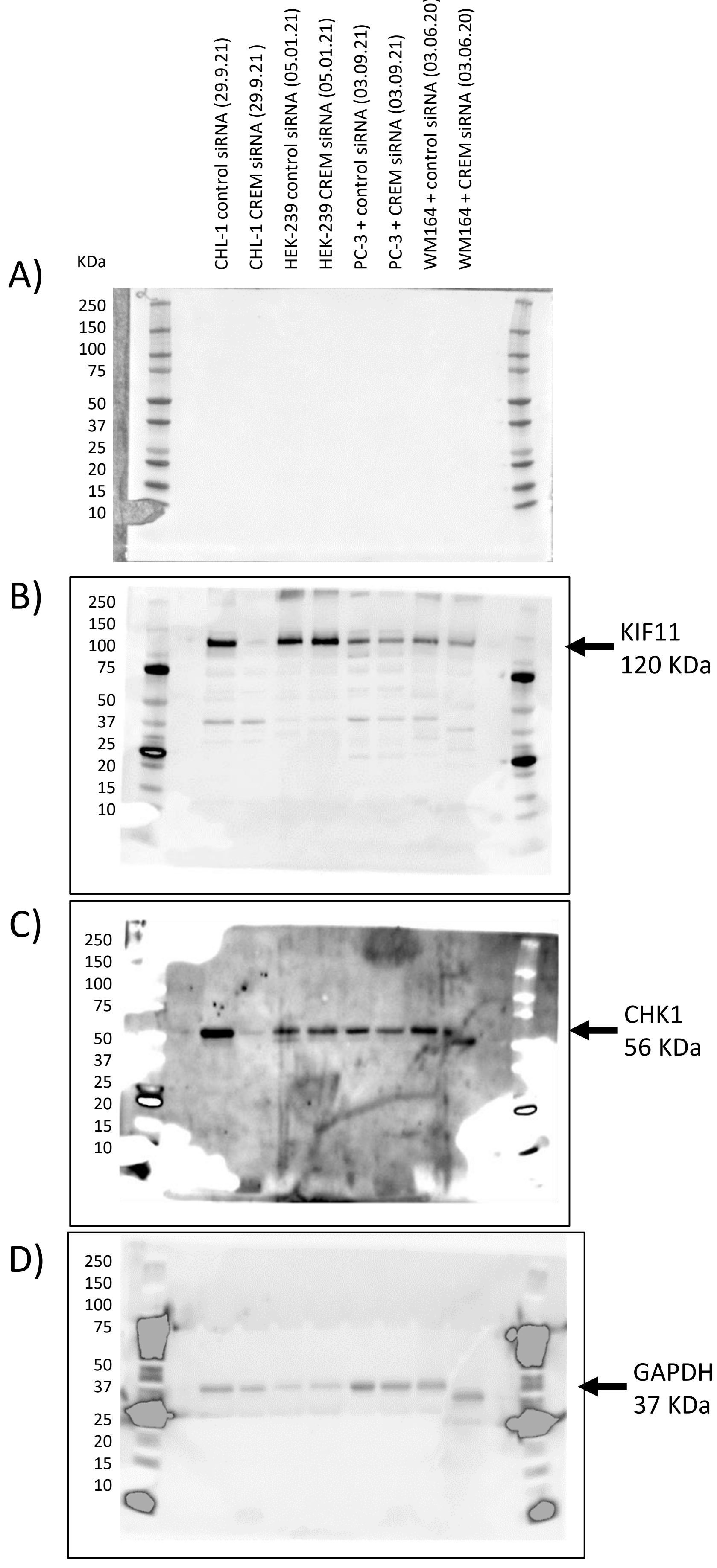

Supplementary Figure 4. The full image of the membrande and ladder (A) used in western blot marked with KIF11 (B; used in Figure 3F and 4E). The same membrane was stripped off and incubated with anti-CHK1 antibody (C; used in Fugure 3F), and GAPDH (D).

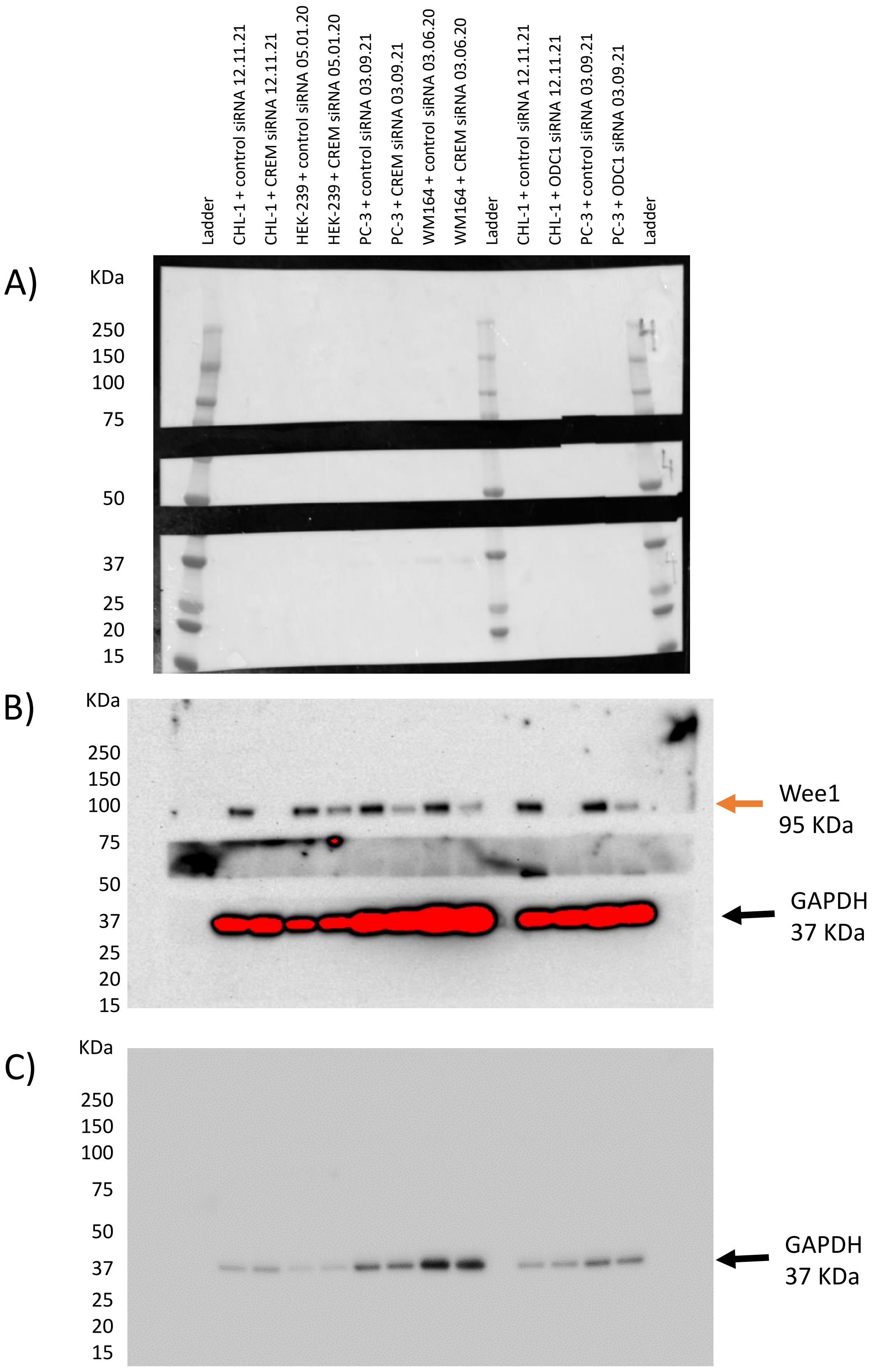

Supplementary Figure 5. The full image of the membrande cut in three pieces (A). Western blot marked with anti-Wee1 (upper part) and GAPDH antibody (lower part) (B; used in Figure 3 F and 4E). Same membrane after shorter exposure showing GAPDH (C; used in Figure 3F).

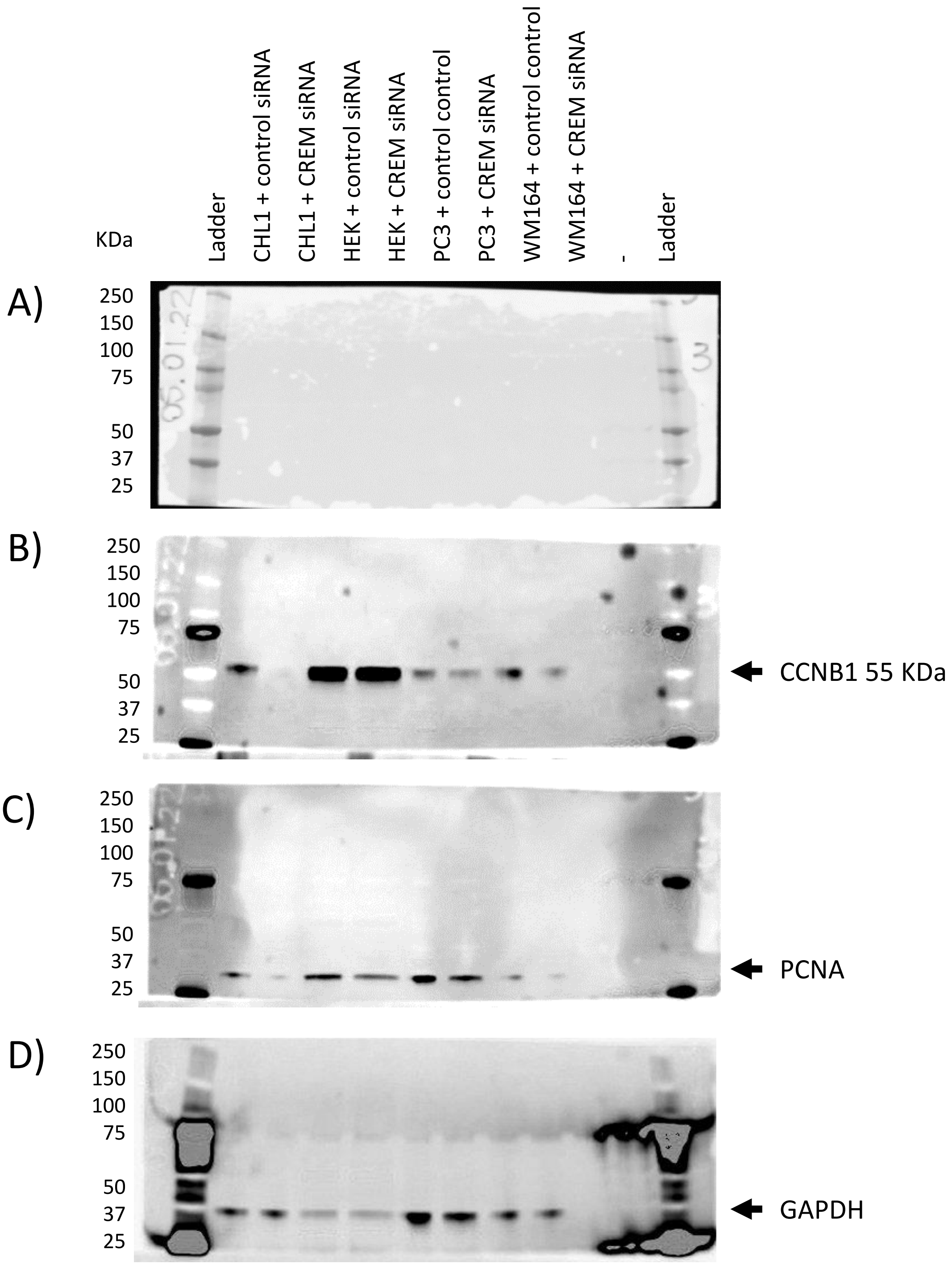

Supplementary Figure 6. The full image of the membrane with ladder (A). Western blot marked with anti-CCNB1 (B; used in Figure 3F and 4E). Membrane was striped off and probed with anti-PCNA (C; used in Figure 3F and 4E) and GAPDH antibody (D) (B; used in Figure 3 F and 4E).

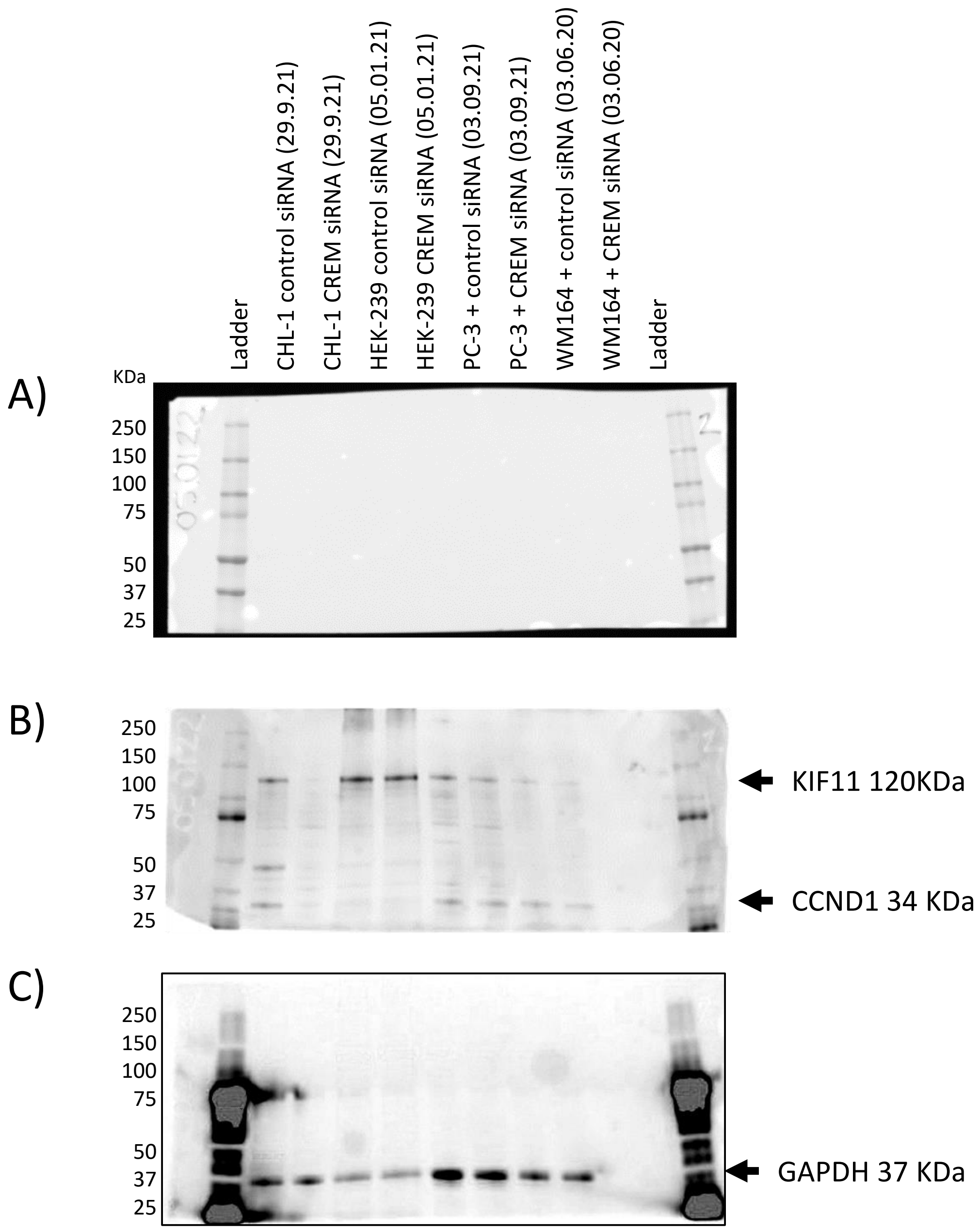

Supplementary Figure 7. The full image of western blot membrane showing the ladder (A). The membrane marked with KIF11 mixed with CCND1 antibody (C; used in Figure 3F). After striped off, the membrane was incubated with anti-GAPDH (B; used in Figure 3F).

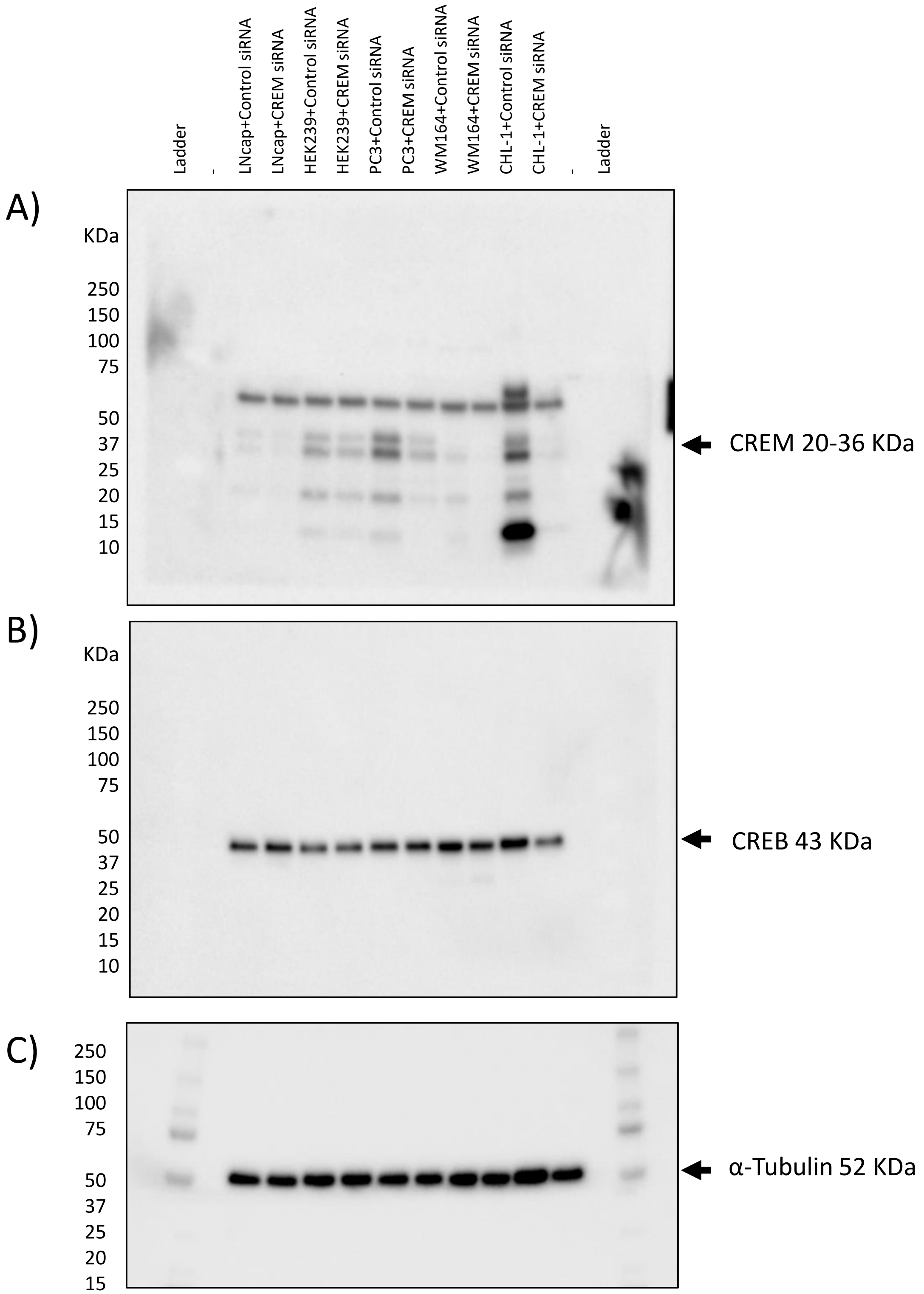

Supplementary Figure 8. The full image of western blot marked with anti-CREM antibody (A). The membrane was stripped off, and marked with anti-CREB (B; used in Figure 4A), and α-Tubulin (C; used in Figure 4A).

A)

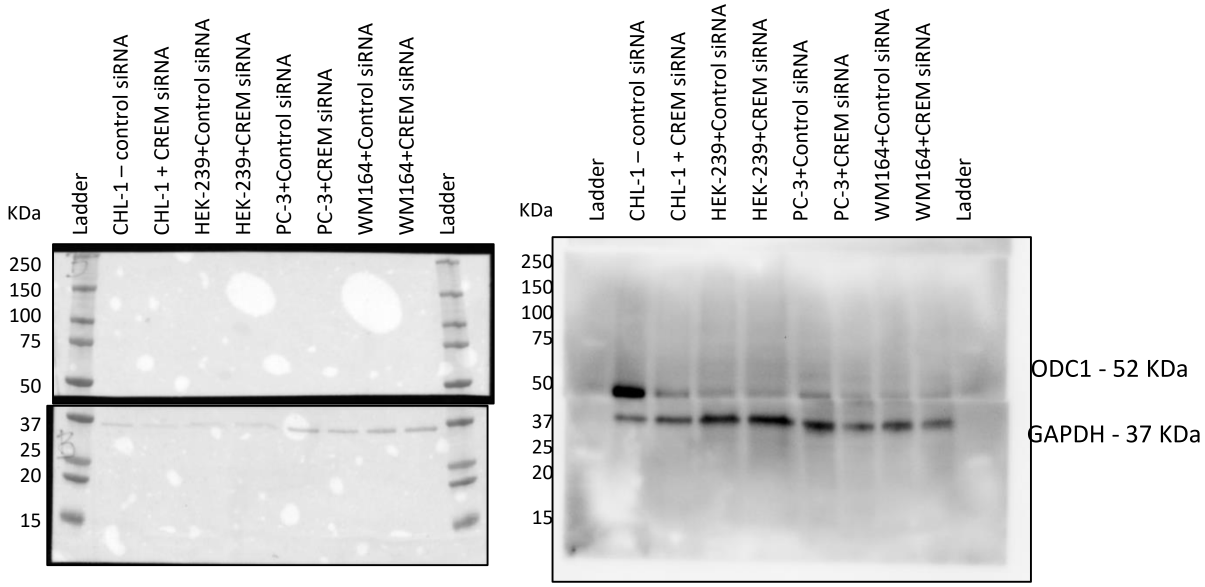

B)

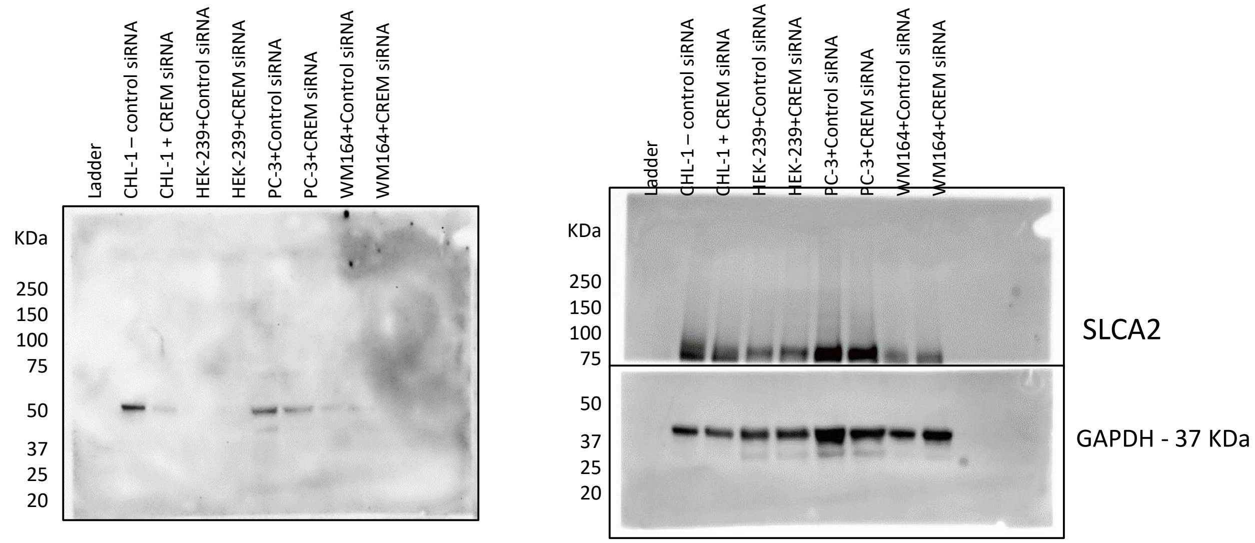

Supplementary Figure 9. The full image of membrane cut in two pieces (A, left) marked with anti-ODC1 and GAPDH antibody (A, right). Membrane market with anti-ODC1 antibody (B, left; used in Figure 5A). The same membrane was cut id two pieces and market with anti- SLCA2 and GAPDH antibody (B, right; GAPDH was used in Figure 5A).

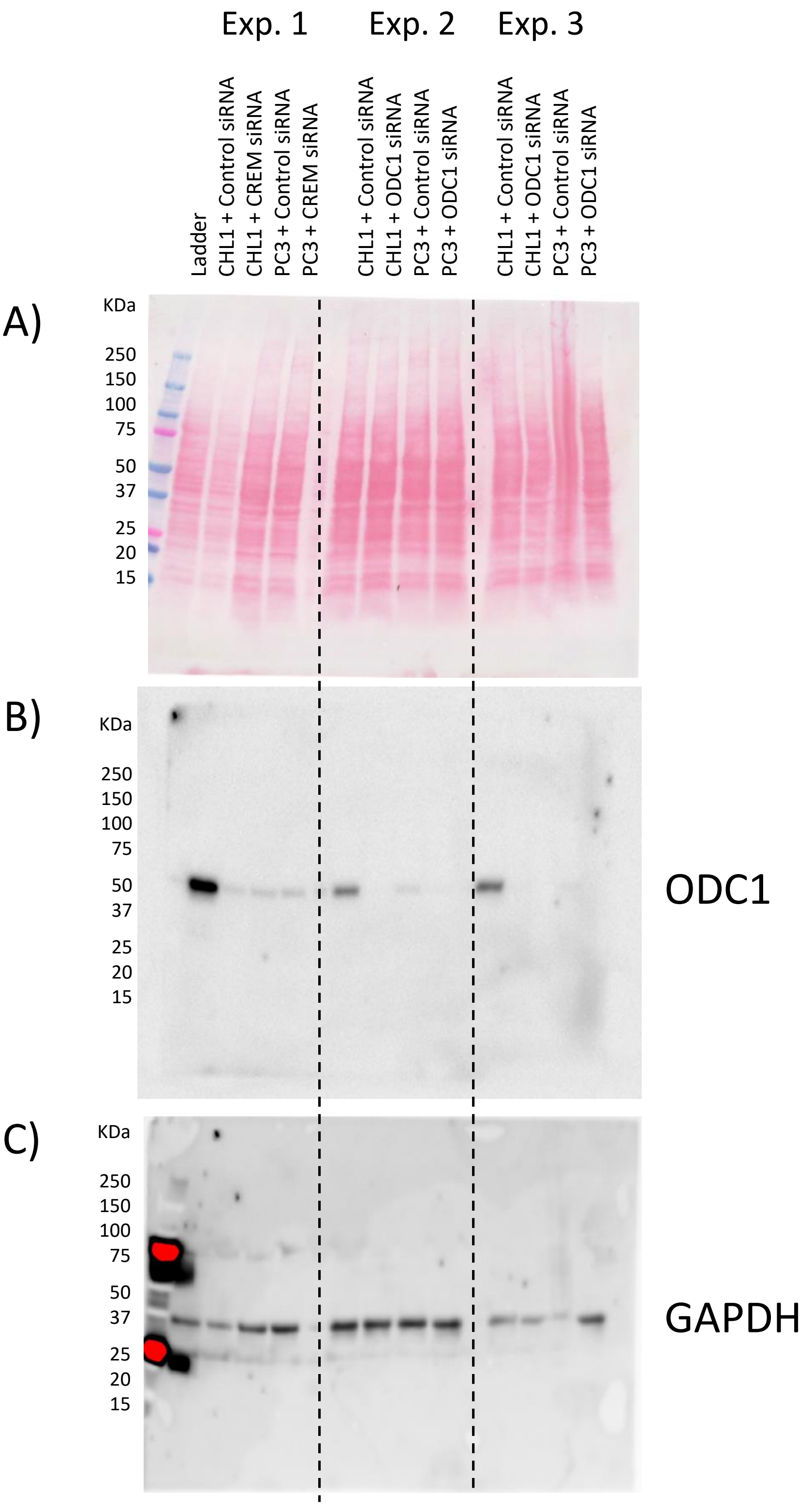

Supplementary Figure 10. The full image of western blot membrane stained with Ponceau showing the ladder (A). The membrane was marked with anti-ODC1 antibody (B). The same membrane was stripped off and incubated with anti-GAPDH antibody to show equal loading (C). Data obtained with Experiment 2 (Exp. 2) was used in Figure 5A.

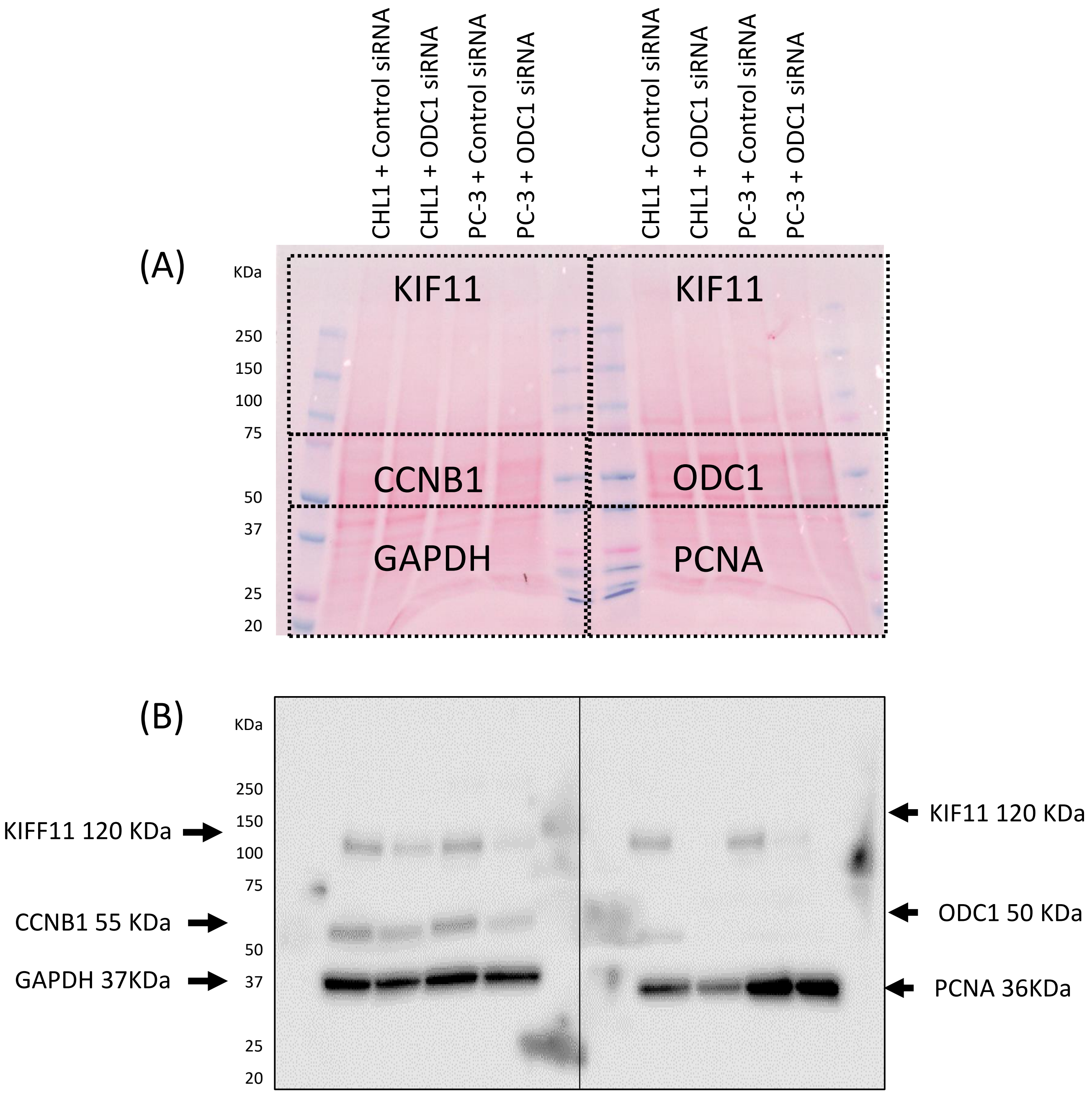

Supplementary Figure 11. The full image of membrane stained with Ponceau showing the ladder and marked the different cut areas that were incubated with anti-KIF11, CCNB1, ODC1, PCNA, or GAPH antibody (B): KIF11, CCNB1, PCNA and GAPDH results were used in Figure 5F.

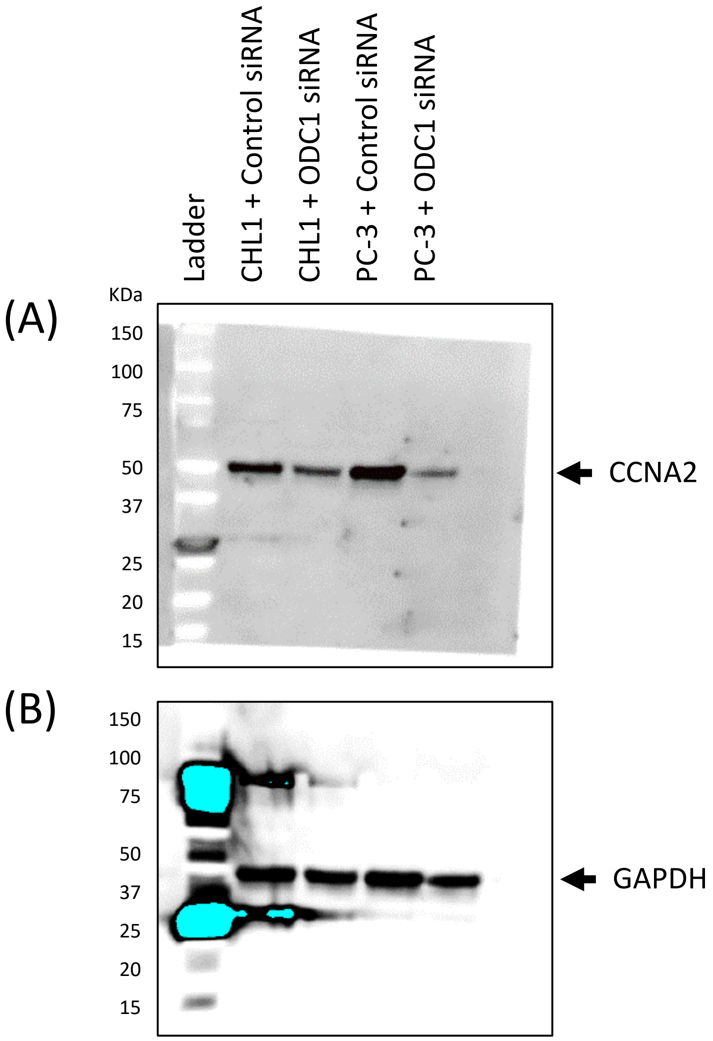

Supplementary Figure 12. The full image of western blot marked with anti-CCNA2 antibody (A; used in Figure Figure 5F). The membrane was stripped off, and marked with anti-GAPDH (B).

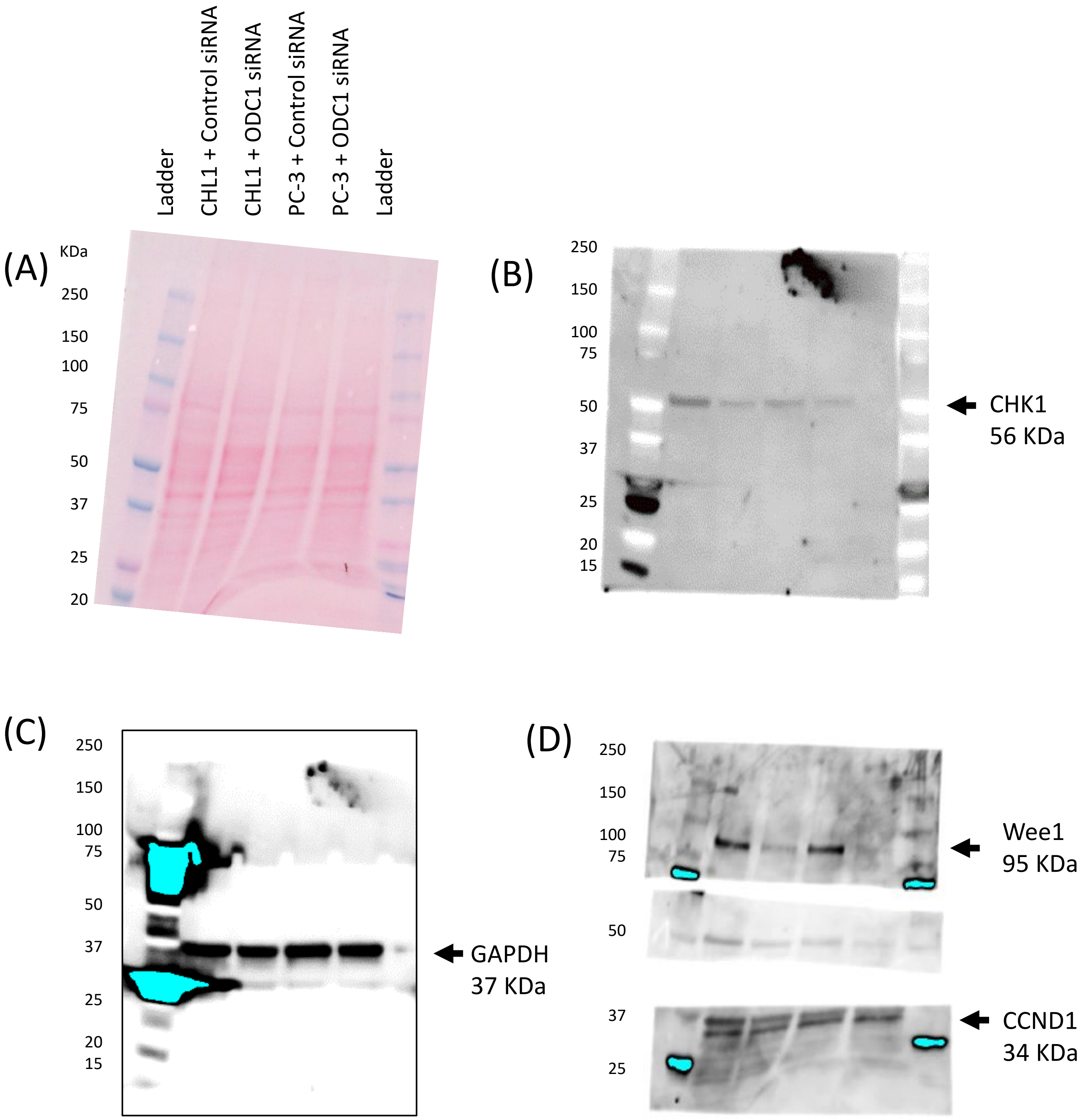

Supplementary Figure 13. The full image of membrane stained with Ponceau and showing the ladder (A). Western blot marked with anti-CHK1 antibody (B; used in Figure 5F), stripped off and incubated with anti-GAPDH antibody(C). The membrane was striped once again and cut to be probed using anti-Wee1 and CCND1 antibodies (D; used in Figure 5F)

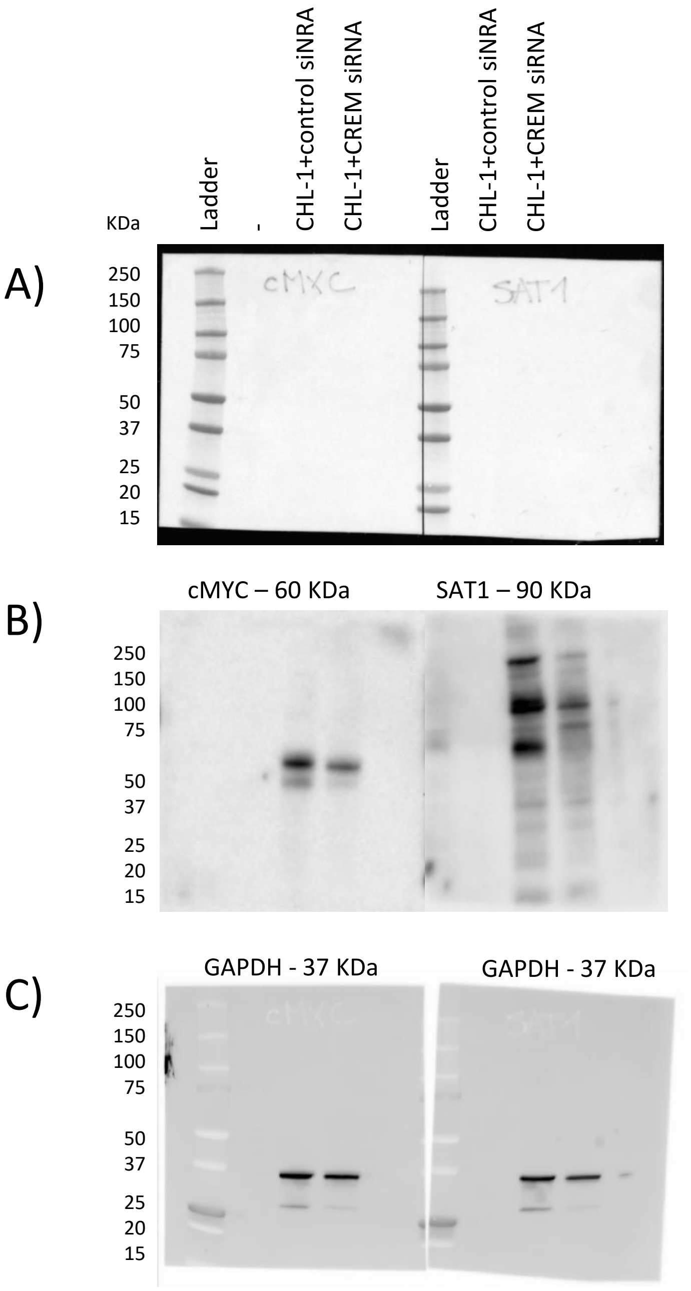

Supplementary Figure 14. The full image of membranes and western blots (A) marked with anti-cMYC (B, left half), and SAT1 antibody (B, right half). Same membrane was stripped off and reprobed using anti-GAPHD antibody (C). Used in Supplent Figure 1).
